# Nitrous oxide inhibition of methanogenesis represents an underappreciated greenhouse gas emission feedback

**DOI:** 10.1093/ismejo/wrae027

**Published:** 2024-03-06

**Authors:** Yongchao Yin, Fadime Kara-Murdoch, Robert W Murdoch, Jun Yan, Gao Chen, Yongchao Xie, Yanchen Sun, Frank E Löffler

**Affiliations:** Center for Environmental Biotechnology, University of Tennessee, Knoxville, TN 37996, United States; Department of Microbiology, University of Tennessee, Knoxville, TN 37996, United States; Biosciences Division, Oak Ridge National Laboratory, Oak Ridge, TN 37831, United States; Center for Environmental Biotechnology, University of Tennessee, Knoxville, TN 37996, United States; Biosciences Division, Oak Ridge National Laboratory, Oak Ridge, TN 37831, United States; Center for Environmental Biotechnology, University of Tennessee, Knoxville, TN 37996, United States; Center for Environmental Biotechnology, University of Tennessee, Knoxville, TN 37996, United States; Department of Microbiology, University of Tennessee, Knoxville, TN 37996, United States; Key Laboratory of Pollution Control and Environmental Engineering, Institute of Applied Ecology, Chinese Academy of Sciences, Shenyang, Liaoning 110016, China; Center for Environmental Biotechnology, University of Tennessee, Knoxville, TN 37996, United States; Department of Civil and Environmental Engineering, University of Tennessee, Knoxville, TN 37996, United States; Center for Environmental Biotechnology, University of Tennessee, Knoxville, TN 37996, United States; Department of Civil and Environmental Engineering, University of Tennessee, Knoxville, TN 37996, United States; Center for Environmental Biotechnology, University of Tennessee, Knoxville, TN 37996, United States; Department of Civil and Environmental Engineering, University of Tennessee, Knoxville, TN 37996, United States; Center for Environmental Biotechnology, University of Tennessee, Knoxville, TN 37996, United States; Department of Microbiology, University of Tennessee, Knoxville, TN 37996, United States; Biosciences Division, Oak Ridge National Laboratory, Oak Ridge, TN 37831, United States; Department of Civil and Environmental Engineering, University of Tennessee, Knoxville, TN 37996, United States; Department of Biosystems Engineering and Soil Science, University of Tennessee, Knoxville, TN 37996, United States

**Keywords:** nitrous oxide, methane, greenhouse gas emissions, inhibition, feedback loop, climate change

## Abstract

Methane (CH_4_) and nitrous oxide (N_2_O) are major greenhouse gases that are predominantly generated by microbial activities in anoxic environments. N_2_O inhibition of methanogenesis has been reported, but comprehensive efforts to obtain kinetic information are lacking. Using the model methanogen *Methanosarcina barkeri* strain Fusaro and digester sludge-derived methanogenic enrichment cultures, we conducted growth yield and kinetic measurements and showed that micromolar concentrations of N_2_O suppress the growth of methanogens and CH_4_ production from major methanogenic substrate classes. Acetoclastic methanogenesis, estimated to account for two-thirds of the annual 1 billion metric tons of biogenic CH_4_, was most sensitive to N_2_O, with inhibitory constants (*K_I_*) in the range of 18–25 μM, followed by hydrogenotrophic (*K_I_*, 60–90 μM) and methylotrophic (*K_I_*, 110–130 μM) methanogenesis. Dissolved N_2_O concentrations exceeding these *K_I_* values are not uncommon in managed (i.e. fertilized soils and wastewater treatment plants) and unmanaged ecosystems. Future greenhouse gas emissions remain uncertain, particularly from critical zone environments (e.g. thawing permafrost) with large amounts of stored nitrogenous and carbonaceous materials that are experiencing unprecedented warming. Incorporating relevant feedback effects, such as the significant N_2_O inhibition on methanogenesis, can refine climate models and improve predictive capabilities.

## Introduction

Carbon dioxide (CO_2_) receives primary attention as a driver for climate change, but methane (CH_4_) and nitrous oxide (N_2_O) account for ~20% and 7%, respectively, of the net radiative forcing in the atmosphere [1, 2]. The central objective of the Paris Agreement, which is to hold the global average temperature increase to “well below 2°C above preindustrial levels” [3, 4], cannot be met without controlling CH_4_ and N_2_O emissions. Global emissions of both CH_4_ and N_2_O are ultimately controlled by microbial processes [2, 5]; however, human activities have massively disturbed the natural balance of microbial production and consumption of these greenhouse gases [6]. The current estimated annual net atmospheric emission increases of ~51 Tg of CH_4_ [7, 8] and 2.2 Tg of N_2_O [9] are both predicted to accelerate [1, 10]. The key microbial guilds and biogeochemical processes responsible for CH_4_ and N_2_O production and consumption are known, and their responses to climate change have been a matter of intense research [11, 12]. One area of considerable uncertainty pertains to positive and negative feedback loops affecting greenhouse gas emissions and climate [13]. An infamous scenario for a positive feedback loop in response to a warming climate is permafrost thawing, which stimulates the microbial activity and the release of CO_2_, CH_4_, and N_2_O from newly bioavailable carbon and nitrogen pools. The massive release of greenhouse gases will further accelerate radiative forcing and in turn cause more thawing. This example illustrates why a comprehensive understanding of both positive and negative feedback loops is essential. Quantitative information is needed to meaningfully incorporate feedback effects into refined climate models.

**Figure 1:**
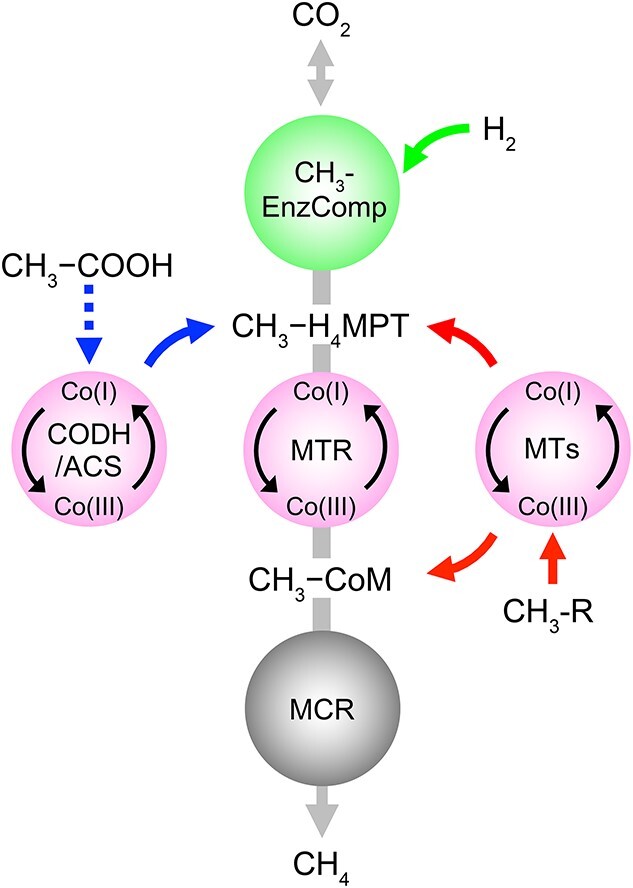
Illustration of the major methanogenic pathways that together account for most of the biogenically produced CH_4_ in nature; acetoclastic (left), hydrogenotrophic (top), and methylotrophic (right) conversions channel into the methanogenic pathway; the central circles indicate steps catalyzed by corrinoid-dependent enzyme systems potentially susceptible to N_2_O inhibition; abbreviations: CH_3_-EnzComp, CH_3_-formation enzyme complexes; CH_3_-H_4_MPT, methyl-tetrahydromethanopterin; MTR, *N*^5^-methyltetrahydromethanopterin:CoM methyltransferase; MTs, substrate-specific methyltransferases; CH_3_-R, methylated compounds (e.g. methanol).

Methanogenic archaea (methanogens) drive CH_4_ production by utilizing acetate, H_2_/CO_2_, and methylated compounds as substrates, generating around 1 billion metric tons of CH_4_ annually [14, 15]. Approximately, two-thirds of biogenic CH_4_ is produced from acetoclastic methanogenesis (i.e. the conversion of acetate to CH_4_ and CO_2_), with the remaining one-third attributed to hydrogenotrophic CO_2_ reduction (hydrogenotrophic pathway) and methylated compound utilization (methylotrophic pathways) [16–20]. While the three major methanogenesis pathways share a core set of enzymes, several mechanistically distinct enzyme systems with corrinoid prosthetic groups (i.e. vitamin B_12_ derivatives) are involved in methyl group transfer reactions, energy conservation, and CH_4_ production (Fig. 1) [18, 19, 21].

In hydrogenotrophic methanogenesis, CO_2_ is sequentially reduced to a methyl group carried by tetrahydromethanopterin (H_4_MPT) with H_2_ as electron donor. The corrinoid-dependent *N*^5^-methyltetrahydromethanopterin:CoM methyltransferase (MTR) complex then transfers the methyl group onto another C_1_-carrier, coenzyme M, forming CH_3_–S–CoM, a step associated with energy conservation, followed by CH_4_ production catalyzed by methyl coenzyme M reductase (MCR) [22]. Acetoclastic methanogens produce CH_4_ by activating acetate to acetyl-CoA, which is then cleaved by the corrinoid-dependent enzyme system carbon monoxide dehydrogenase/acetyl-CoA synthase (CODH/ACS) to yield an enzyme-bound methyl group and a carbonyl group. The carbonyl moiety is oxidized to CO_2_ and the methyl group is transferred to H_4_MPT [23]. The MTR complex catalyzes a methyl group transfer to form CH_3_–S–CoM, a step associated with energy conservation, before MCR mediates the reduction of the methyl group to CH_4_ [16, 24]. Methylotrophic methanogens either directly generate CH_3_–S–CoM from methylated compounds (e.g. MeOH) utilizing substrate-specific, corrinoid-dependent MTRs, or cleave the methyl group from methoxylated compounds (e.g. 2-methoxybenzoate) and form CH_3_–CoM via H_4_MPT and the MTR complex [25], followed by CH_4_ production via MCR. All three methanogenesis pathways utilize corrinoid-dependent enzyme systems for methyl group transfers and have strict requirements for super-reduced Co(I) to generate CH_4_ and conserve energy [26, 27]. These features render methanogenesis sensitive to oxidative stress (e.g. fluctuating redox conditions and oxygen intrusion) [19, 28].

N_2_O is an even stronger oxidant than oxygen (*E*^o^’(N_2_O(g)/N_2_(g) = +1.355 V > *E*^o^’(O_2_(g)/H_2_O(l) = +0.818 V), reacts with Co(I), and has been shown to interfere with Co(I) cobamide-dependent enzyme systems [29, 30]. Demonstrated metabolic consequences include ceased corrinoid-dependent methionine biosynthesis and impaired organohalide respiration [31–33]. Based on this information, elevated N_2_O in anoxic ecosystems would be expected to inhibit other corrinoid-dependent processes such as methanogenesis; however, available studies investigating the impacts of N_2_O on methanogenesis have led to inconsistent N_2_O inhibition patterns for axenic methanogen cultures and CH_4_ producing microbial communities [34–36]. Laboratory incubations showed that *Methanobacterium bryantii* strain Bab1 grown with H_2_/CO_2_ ceased CH_4_ production in the presence of 95 μM N_2_O, whereas *Methanosarcina barkeri* strain MS maintained some methanogenic activity at 10-fold higher N_2_O concentrations under the same growth conditions [35]. Different sensitivities to N_2_O inhibition were also reported for mixed methanogenic cultures maintained with different substrates. For example, inhibition of CH_4_ production in a mixed community bioreactor occurred only at N_2_O concentrations exceeding 700 μM [36], whereas 20–28 μM N_2_O completely inhibited methanogenic activity in salt marsh sediment and Amazon peatland enrichment cultures [34, 37]. These variable sensitivities to N_2_O suggest that inhibition of methanogenesis by N_2_O is organism- and possibly substrate-specific; however, the available data are scarce and do not allow a robust, quantitative assessment of N_2_O inhibition on methanogenesis from relevant methanogenic substrates [34–36].

N_2_O fluxes in soil–water systems have risen sharply due to the intensified use of synthetic N fertilizer in agriculture [6, 9, 38]. As a result, elevated N_2_O concentrations occur more frequently in ecosystems with CH_4_ production such as rice paddy soils [39], wastewater treatment plants [40], sediments [41], and groundwater aquifers [42]. Also, permafrost thawing accelerates N turnover, releasing large amounts of N_2_O [38, 43]. More detailed knowledge about the interactions between N_2_O concentrations and methanogenesis is needed to advance the predictive capabilities of climate change impacts on future greenhouse gas emission scenarios. To address the existing knowledge gaps, we assessed the inhibitory effect of N_2_O on CH_4_ production from major methanogenic substrates in growth experiments with axenic and mixed methanogenic cultures and in whole-cell suspension assays. Using cultures performing acetoclastic, methylotrophic, and/or hydrogenotrophic methanogenesis, we determined kinetic parameters that quantitatively describe N_2_O inhibition on methanogenesis.

## Materials and methods

### Methanogenic cultures and growth inhibition experiments

The methanogenic archaeon *M. barkeri* strain Fusaro metabolizes acetate, H_2_/CO_2_, and MeOH while employing different, substrate-specific methanogenic pathways. To determine if *M. barkeri* exhibits varied sensitivities to N_2_O, cultures were pregrown with MeOH, H_2_/CO_2_, or acetate for at least three consecutive transfers before the impact of N_2_O on CH_4_ production was examined. Also, we analyzed three methanogenic mixed cultures derived from anaerobic digester sludge, which is known to harbor a broad diversity of methanogenic archaea. Three different enrichment cultures from the same source material were obtained with MeOH, H_2_/CO_2_, or acetate as growth substrate. The mixed cultures were transferred at least six times on the respective substrate and were used to examine the impact of N_2_O on CH_4_ production from different methanogenic substrates (see Supplemental Information for additional information on the mixed methanogenic cultures). Experiments were performed in triplicate 60-ml glass serum bottles with 30 ml N_2_/CO_2_ (80/20, v/v) headspace and 30 ml bicarbonate-buffered (50 mM) mineral salt medium (pH 7.2) reduced with 0.2 mM sulfide and 0.2 mM L-cysteine [44]. Acetoclastic, methylotrophic, and hydrogenotrophic cultures received 20 mM acetate, 30 mM MeOH, and 1.24 mmol H_2_, respectively. To avoid overpressure in bottles with H_2_ as electron donor, the headspace of culture bottles was replaced with 30 ml of filter-sterilized H_2_. For *M. barkeri* cultures, 0.1–1.0 ml of N_2_O gas (undiluted or 10-fold diluted in N_2_) was directly added to the incubation vessels to achieve final aqueous N_2_O concentrations of 100 and 200 μM in cultures with MeOH, 50 and 100 μM in cultures with H_2_, and 20 and 50 μM in cultures with acetate. For the methanogenic mixed cultures grown with 30 mM MeOH, 30 ml H_2_ (1.24 mmol), and 20 mM acetate, 0.5–1.6 ml of 10-fold diluted N_2_O gas (in N_2_) was introduced to achieve final aqueous phase N_2_O concentrations of 10 and 30 μM. More detailed information about N_2_O additions and concentration calculations is provided in the Supplemental Methods. All cultures were incubated without agitation at 37°C in the dark with the stoppers facing up, and replicates without N_2_O and without inoculum served as positive and negative controls, respectively. CH_4_ and N_2_O were analyzed throughout the growth experiments by injecting 100 μl headspace samples into an Agilent 3000A Micro gas chromatograph equipped with thermal conductivity detectors and a molecular sieve column and a PLOT Q column for CH_4_ and N_2_O measurements, respectively.

### Whole-cell suspension assays to determine N_2_O inhibition constants for CH_4_ production

The *M. barkeri* and the methanogenic mixed cultures were first grown in 1.6 l medium and harvested via centrifugation when about two-thirds of the initial substrate (i.e. MeOH, H_2_/CO_2_, or acetate) had been converted to CH_4_, as calculated based on CH_4_ production according to Equations (1)–(3) (see below). The cell pellets collected from 1.6 l of medium were washed and suspended in 1.6 ml of reduced mineral salt medium in sealed 2-ml glass vials, resulting in a 1000-fold concentration of the biomass. A 0.2 ml aliquot of the concentrated cell suspension was sacrificed to measure total protein with the Bradford assay [45].

Cell suspension assays were performed at room temperature in 20-ml glass vials flushed with N_2_/CO_2_ (80/20, v/v) and were sealed with Teflon-lined butyl rubber stoppers held in place with aluminum crimps. The assay vials received a total of 0.9 ml reduced mineral salt medium, 0.1 ml of cell suspension, and increasing concentrations of substrates (i.e. MeOH, H_2,_ or acetate, with N_2_O as indicated in Tables S1–S7). For assay vials receiving H_2_ as electron donor, the headspace was replaced with increasing volumes of premixed H_2_/CO_2_ (4/1, v/v) to achieve H_2_ concentrations ranging from 1.2 to 333 μM (Table S3). Small volumes (89–178 μl) of undiluted or 10-fold diluted (in N_2_) N_2_O were directly injected into the 20-ml assay vials, what resulted in small pressure changes with negligible impact on the distribution of N_2_O between the aqueous phase and the headspace. All cell suspensions were freshly prepared following identical procedures to ensure consistency between independent experiments. Vials that received 0.1 ml of sterile mineral salt medium or 0.1 ml of heat-killed (i.e. autoclaved) cell suspension served as negative controls.

Following 10 min of equilibration, 0.1 ml of cell suspension was added to initiate the assays. Headspace samples (100 μl) were withdrawn with an air-tight syringe with a lock every 30 min over a 3-h incubation period and, for the kinetic experiments, CH_4_ was analyzed using an Agilent 7890 GC series gas chromatograph equipped with a flame ionization detector and a DB-624 capillary column (60 m length × 0.32 mm diameter, 1.8 μm film thickness). For each treatment at a fixed initial substrate concentration [S], an initial CH_4_ production rate *v*, normalized to the amount of protein per vial in the unit of nmol CH_4_ min^−1^ mg protein^−1^, was determined. The determined rate data were fit into Michaelis–Menten competitive, noncompetitive, and uncompetitive inhibition models (Table S8) to determine the maximum CH_4_ production rate *V*_max_, the half-velocity constant *K_m_*, and the inhibitory constant *K_I_* of N_2_O on CH_4_ production from the different substrates. The best-fit inhibition model was chosen based on the highest coefficient of determination (*R*^2^) and the lowest standard deviation of the residuals (Table S9). Each datum point on the Michaelis–Menten plots (Figure 5) represents a CH_4_ production rate generated from at least four time points for one substrate concentration [S].

### Genomic DNA extraction and 16S ribosomal RNA gene amplicon sequencing

DNA extraction and PCR assays followed established procedures [46] and details are provided in the Supplemental Information. The purified DNA samples were processed and barcoded with primers 341F/785R targeting the V3/V4 region of the prokaryotic 16S rRNA gene [47] following established procedures [48]. The resulting sequence data were analyzed using the QIIME 2 v2021.4 environment [49]. The precise programs and settings are described in the Supplemental Information, and the QIIME 2 pipeline script and custom R file employed to parse results are available at https://github.com/rwmurdoch/methanogens_and_N2O. The raw amplicon library reads were deposited in the Sequence Read Archive (SRA) under accessions SRR19782291 to SRR19782296.

### Quantitative real-time PCR and growth yield calculations

Quantitative real-time PCR (qPCR) to enumerate archaeal 16S rRNA genes in *M. barkeri* and methanogenic mixed cultures followed established protocols using primer set Mtgen835F/918R and probe FAM-Mtgen831 (Table S10) [50]. Samples for enumeration of cell numbers were collected at the beginning and at the end of the prolonged growth experiments. Average growth yields of methanogens were calculated from the changes in 16S rRNA gene copy numbers in triplicate culture vessels divided by the total amounts of CH_4_ produced over the same time period (Table S11). Reported growth yield (i.e. cells produced per μmol of CH_4_ formed) used conversion factors of 3 and 2.5 16S rRNA gene copies per methanogen cell for *M. barkeri* [51] and the enrichment cultures [52], respectively. For comparison with theoretical [53] and reported values from the literature (Table 2), growth yields were also calculated as μg of dry biomass per μmol of CH_4_ formed making the following assumptions: an average methanogen cell has a volume of 2.5 μm^3^ [54] with a density equal to water [55]. The dry cell biomass is 30% of the wet cell biomass [56], and 90% of the dry biomass represents organic material [55].

**Table 1:** Kinetic (*V*_max_, *K_m_*) and inhibition (*K_I_*) parameters for CH_4_ production from MeOH, H_2_, and acetate determined in concentrated whole-cell suspension assays of *M. barkeri* and methanogenic mixed cultures in response to increasing N_2_O concentrations.^a^

Culture	Substrate	N_2_O (μM)	*V*_max_ (nmol CH_4_ min^−1^ mg protein^−1^)	*K_m_* (μM)	*K_I_* (μM)
*M. barkeri*	MeOH	0	440.4 ± 10.2	2.1 (±0.1) × 10^3^	130.9 ± 4.7
		100	244.1 ± 38.4		
		200	188.8 ± 32.5		
*M. barkeri*	H_2_	0	138.1 ± 6.9	51.1 ± 7.2	90.6 ± 10.8
		50	82.8 ± 29.5		
		100	59.9 ± 5.9		
*M. barkeri*	Acetate	0	40.8 ± 4.7	13.4 (±2.7) × 10^3^	24.8 ± 3.1
		20	32.1 ± 4.2		
		50	22.8 ± 2.5		
Mixed culture	MeOH	0	209.0 ± 4.4	359.3 ± 30.8	109.9 ± 6.8
		50	130.8 ± 22.6		
		100	51.4 ± 44.7		
Mixed culture	H_2_	0	105.8 ± 3.7	19.0 ± 2.3	62.1 ± 6.4
		30	71.6 ± 10.4		
		60	36.6 ± 13.6		
Mixed culture	Acetate	0	22.8 ± 0.7	302.2 ± 46.2	17.7 ± 1.8
		10	14.1 ± 1.8		
		30	5.7 ± 3.6		

^a^Data listed show results from the best fit Michaelis-Menten single-substrate-single-inhibitor models. Error values represent 95% confidence intervals.

**Table 2:** Comparison of growth yields of methanogens utilizing different substrates with values collected from the peer-reviewed literature and calculated values based on thermodynamics and bioenergetic principles.

			Growth yield (μg organic matter per μmol CH_4_ formed)		
Cultures	Substrates	N_2_O (μM)	Measured^a^	Predicted from thermodynamics^b^	Range (references)
*M. barkeri*	MeOH	0	3.57 ± 0.32	5.2	3.3–6.4 ([71, 72])
	MeOH	100	1.30 ± 0.28		
	MeOH	200	0.62 ± 0.03		
	H_2_	0	0.53 ± 0.14	3.6	5.4–8.6 ([73])
	H_2_	50	0.15 ± 0.02		
	H_2_	100	0.08 ± 0.02		
	Acetate	0	1.45 ± 0.46	2.1	1.4–5.7 ([74])
	Acetate	20	1.02 ± 0.16		
	Acetate	50	0.70 ± 0.02		
Mixed methanogenic cultures	MeOH	0	4.30 ± 0.56	5.2	3.3–6.4 ([72])
	MeOH	10	0.14 ± 0.05		
	MeOH	30	NA		
	H_2_	0	0.43 ± 0.05	3.6	1.3–7.2 ([14])
	H_2_	10	0.16 ± 0.01		
	H_2_	30	NA		
	Acetate	0	0.65 ± 0.05	2.1	1.4–5.7 ([23, 64, 75])
	Acetate	10	0.31 ± 0.02		
	Acetate	30	NA		

a Cell numbers were determined with qPCR. Because *M. barkeri* has three copies of the 16S rRNA gene, the qPCR results were divided by a factor of three to obtain cell numbers. Calculation of methanogen cell numbers in the mixed cultures assumed an average 16S rRNA gene content of 2.5.

b Theoretical values calculated in this study based on thermodynamics and published information [53].

## Results

### N_2_O has distinct impact on CH_4_ production from different methanogenic substrates

**Figure 2:**
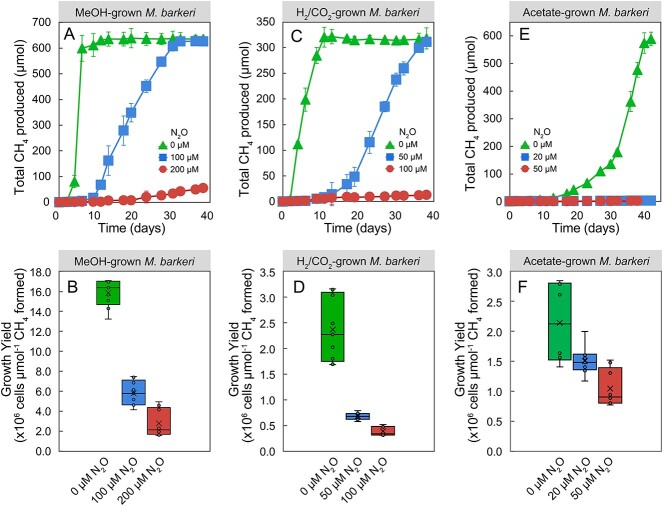
Effect of N_2_O on CH_4_ production and growth yields in axenic *M. Barkeri* cultures; the upper panels show time courses of CH_4_ production in cultures that received MeOH (A), H_2_ (C), or acetate (E); the bottom panels display growth yields after 38-day incubation for *M. Barkeri* growing with MeOH (B), H_2_ (D), or acetate (F); error bars represent the standard deviation of replicate samples and are not shown when smaller than the symbol size; *n* = 3 for (A), (C), and (E); *n* = 9 (including three technical replicates for triplicate biological samples) for (B), (D), and (F).

In the absence of N_2_O, MeOH-grown *M. barkeri* cultures consumed 895 ± 10 μmol of MeOH within a 6-day incubation period and produced 635 ± 34 μmol of CH_4_ (Fig. 2A). This stoichiometry closely matched the expected CH_4_ production based on Equation (1): 


(1) \begin{equation*} {4\mathrm{CH}}_3\mathrm{OH}\to{3\mathrm{CH}}_4+{\mathrm{CO}}_2+{2\mathrm{H}}_2\mathrm{O}\kern0.75em {\Delta \mathrm{G}}^{\mathrm{o}}=\hbox{--} 105\ \mathrm{kJ}\ {\mathrm{mol}}^{\hbox{--} 1}{\mathrm{CH}}_4 \end{equation*}

Growth yields of 5.3 × 10^6^ ± 0.3 × 10^6^ cells per μmol CH_4_ were measured for cultures without N_2_O (Fig. 2B). By contrast, cultures that received 100 or 200 μM N_2_O produced negligible amounts of CH_4_ during the initial 6-day incubation period without any apparent growth after 6 days. Following an 11-day lag phase, cultures with 100 μM N_2_O started consuming MeOH, and 626 ± 20 μmol of CH_4_ were produced following a 38-day incubation period, indicating that 100 μM N_2_O delayed, but did not prevent, CH_4_ production from MeOH by *M. barkeri* (Fig. 2A). Although complete conversion of MeOH to CH_4_ according to Equation (1) was achieved in the presence of 100 μM N_2_O over a prolonged 38-day incubation period, the growth yield decreased by 63.8% ± 7.8% compared to cultures without N_2_O, (i.e. 1.9 × 10^6^ ± 0.4 x 10^6^ vs. 5.3 × 10^6^ ± 0.3 × 10^6^ cells were produced per μmol of CH_4_ formed) (Fig. 2B). In the presence of 200 μM N_2_O, only 55 ± 11 μmol of CH_4_ were produced over a 38-day incubation period, and the growth yield decreased by over 80% to 0.9 × 10^6^ ± 0.4 × 10^6^ cells per μmol CH_4_, indicating a pronounced inhibitory effect of N_2_O on CH_4_ production and growth of *M. barkeri*.

More pronounced N_2_O inhibition on CH_4_ production and growth was observed in *M. barkeri* cultures that received H_2_ as electron donor (i.e. hydrogenotrophic methanogenesis). In the absence of N_2_O, *M. barkeri* cultures, that received H_2_ as electron donor, produced 318 ± 21 μmol of CH_4_ from 1.24 mmol of H_2_ over an 11-day incubation period, consistent with Equation (2): 


(2) \begin{equation*} {4\mathrm{H}}_2+{\mathrm{CO}}_2\to{\mathrm{CH}}_4+{2\mathrm{H}}_2\mathrm{O}\kern0.5em {\Delta \mathrm{G}}^{\mathrm{o}}=\hbox{--} 131\ \mathrm{kJ}\ {\mathrm{mol}}^{\hbox{--} 1}{\mathrm{CH}}_4 \end{equation*}

In the absence of N_2_O, a growth yield of 0.8 × 10^6^ ± 0.2 × 10^6^ cells per μmol of CH_4_ formed was measured (Fig. 2C and D). In the presence of 50 or 100 μM N_2_O, CH_4_ production by *M. barkeri* cultures commenced after prolonged lag phases ranging from 13 to 17 days. At the end of the 38-day incubation period, *M. barkeri* cultures with 50 μM N_2_O and produced 311 ± 7 μmol of CH_4_. The *M. barkeri* cultures that had received 100 μM N_2_O only generated 17 ± 3.2 μmol of CH_4_, resulting in a 95% decrease in total CH_4_ production compared to cultures without N_2_O. The average growth yield in cultures with 50 or 100 μM N_2_O decreased by ~70% and 85% to 2.3 × 10^5^ ± 0.3 × 10^5^ and 1.2 × 10^5^ ± 0.3 × 10^4^ cells per μmol CH_4_ formed, respectively, compared to cultures without N_2_O.

The most pronounced N_2_O inhibition was observed in acetate-fed *M. barkeri* cultures, and 20 μM N_2_O severely diminished CH_4_ production (Fig. 2E). In the absence of N_2_O, *M. barkeri* produced 588 ± 24 μmol of CH_4_ from 605 ± 17 μmol of acetate over a 38-day incubation period, consistent with Equation (3): 


(3) \begin{equation*} {\mathrm{CH}}_3{\mathrm{CO}\mathrm{O}}^{-}+{\mathrm{H}}^{+}\to{\mathrm{CH}}_4+{\mathrm{CO}}_2\ {\Delta \mathrm{G}}^{\mathrm{o}}\hbox{'}=\hbox{--} 35\ \mathrm{kJ}\ {\mathrm{mol}}^{\hbox{--} 1}\ {\mathrm{CH}}_4 \end{equation*}

In the presence of 20 or 50 μM N_2_O, *M. barkeri* cultures only produced 21.3 ± 4.1 and 20.4 ± 3.8 μmol of CH_4_, respectively, a decline of over 96% in total CH_4_ production compared to cultures without N_2_O over the 38-day incubation period. Acetate-grown *M. barkeri* cultures that had received 20 or 50 μM N_2_O generated 1.5 ± 0.2 × 10^6^ and 1.0 ± 0.3 × 10^6^ cells per μmol of CH_4_ produced, decreases of 30.4% ± 3.3% and 52.5% ± 12.6%, respectively, compared to the average yield of 2.1 × 10^6^ ± 0.7 × 10^6^ cells per μmol CH_4_ in cultures without N_2_O (Fig. 2F).

In all culture vessels with observed inhibition of methanogenic activity, N_2_O concentrations remained constant throughout the experiment, consistent with the absence of a “*nos*” operon on the genome of *M. barkeri*. Taken together, these results demonstrate that micromolar levels of N_2_O inhibit methanogenesis, reduce growth yields of this model methanogen, and further reveal that the inhibition is most pronounced for acetoclastic methanogenesis.

### N_2_O adversely affects CH_4_ production and methanogen growth yields in mixed methanogenic enrichment cultures

**Figure 3:**
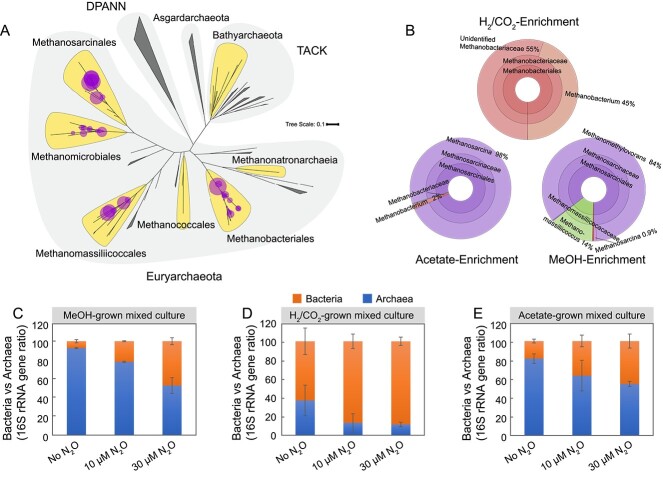
The composition and relative abundances of total sequences representing methanogenic archaea in mixed cultures enriched with acetate, H_2_/CO_2_, or MeOH; (A) phylogenetic placements of archaeal 16S rRNA gene amplicons detected in the enrichment cultures; the highlighted lobes indicate major clades of archaea known or suspected to produce CH_4_; the circles indicate best phylogenetic placements of archaeal taxa identified across all enrichment conditions; the size of the circle is proportional to the number of actual sequence variants (ASVs) detected; large shaded areas indicate archaeal superphyla, including *Diapherotrites*, *Parvarchaeota*, *Aenigmarchaeota*, *Nanohaloarchaeota*, and *Nanoarchaeota* (DPANN) and *Thaumarchaeota*, *Aigarchaeota*, *Crenarchaeota*, and *Korarchaeota* (TACK); see Supplemental Information for details on tree construction and fragment placement methodology; (B) relative abundances of total sequences representing methanogenic archaea in mixed cultures; panels (C) MeOH, (D) H_2_/CO_2_, and (E) acetate depict qPCR data showing the proportional changes of total bacterial and total archaeal (methanogen) 16S rRNA genes in the mixed cultures without N_2_O and in the presence of 10 and 30 μM N_2_O; error bars represent the standard deviation of replicate samples (*n* = 9, three technical replicates of triplicate biological samples).

To examine the impact of N_2_O on mixed methanogen communities, growth and kinetic assays were performed with enrichment cultures derived from digester sludge. Microbial community analysis revealed the presence of diverse methanogen groups known to utilize acetate, H_2_/CO_2_, and MeOH as substrates, with distinct methanogen taxa prevalent under the different enrichment conditions (Tables S12 and S13). In cultures that received H_2_ as electron donor, sequences representing the genus *Methanobacterium* and other unidentified members of the family *Methanobacteriaceae* dominated, whereas in acetate-fed cultures, *Methanosarcina* was the most abundant archaeal taxon. *Methanomethylovorans* was the most abundant archaeal genus in the MeOH-fed cultures, but sequences representing the genus *Methanomassiliicoccus* were also prevalent. 16S rRNA gene amplicons representing six families and four of the known eight orders of methanogens were represented in the examined mixed cultures derived from digester sludge (Fig. 3A and B; Table S12).

**Figure 4:**
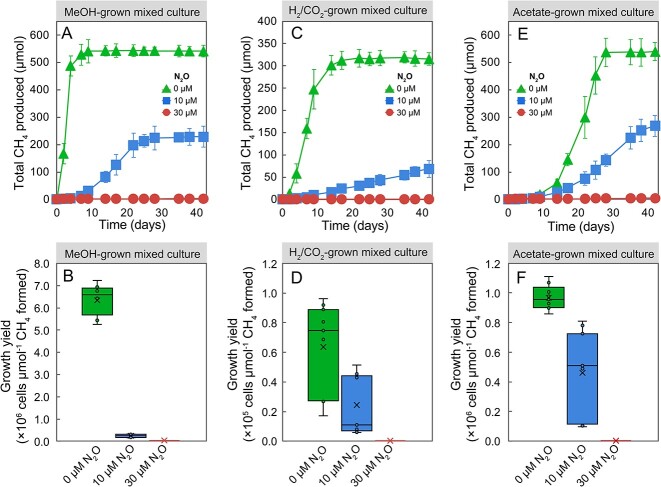
Effects of N_2_O on CH_4_ production and growth yields in methanogenic mixed cultures enriched with MeOH, H_2_, or acetate; the upper panels depict CH_4_ production from MeOH (A), H_2_ (C), and acetate (E); the bottom panels demonstrate methanogen growth yield differences in cultures amended with MeOH (B), H_2_ (D), or acetate (F); error bars represent standard deviation and are not shown when smaller than the symbol size (*n* = 3 for upper panels; *n* = 9 for bottom panels [three technical replicates of triplicate biological samples]).

Without N_2_O addition, the mixed cultures produced 542.6 ± 23.3, 316.5 ± 25.4, and 589.2 ± 33.7 μmol of CH_4_ from 0.9 mmol of MeOH, 1.24 mmol of H_2_, or 0.6 mmol of acetate, consistent with Equations (1)–(3). When cultures were amended with 10 or 30 μM N_2_O, CH_4_ production in MeOH-, H_2_-, and acetate-fed methanogenic mixed cultures was substantially or completely inhibited (Fig. 4A, C, and E). Even over an extended 42-day incubation period, the presence 10 μM N_2_O still repressed the total CH_4_ production in MeOH-, H_2_-, or acetate-grown mixed cultures by ~60%, 80% and 50%, respectively, compared to incubations without N_2_O. With 30 μM N_2_O, only negligible amounts of CH_4_ were detected in all incubations over a 42-day incubation period. Some N_2_O loss was observed in the mixed culture vessels that received H_2_/CO_2_ or MeOH as substrates, with no more than 20% of the initial amount of N_2_O consumed. Taken together, these results demonstrate that N_2_O exerts a stronger inhibitory effect on methanogenesis in the mixed cultures harboring diverse methanogen populations than in axenic *M. barkeri* incubations.

**Figure 5:**
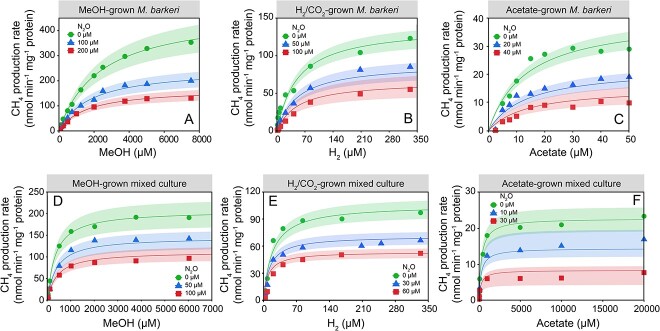
Kinetics of CH_4_ production from MeOH, H_2_, and acetate in whole-cell suspension assays of *M. Barkeri* and the methanogenic mixed cultures in the presence of increasing concentrations of N_2_O; the upper panels show the Michaelis–Menten plots of CH_4_ production rates versus the respective substrate concentrations in cell suspensions of *M. Barkeri* without and in the presence of increasing N_2_O concentrations in basal salt medium amended with MeOH (A), H_2_ (B), or acetate (C); the bottom panels show Michaelis–Menten plots of CH_4_ production rates versus the respective substrate concentrations in concentrated whole-cell suspensions of the methanogenic mixed cultures without N_2_O and in the presence of increasing N_2_O levels in basal salt medium amended with MeOH (D), H_2_ (E), or acetate (F); the shaded ribbons represent the standard distances (95% confidence interval) between the measured values and the nonlinear regression lines.

Enumeration of total methanogens and total bacteria in the mixed cultures using qPCR revealed that N_2_O diminished methanogen growth yields (Fig. 4B, D, and F). The qPCR analysis further revealed significantly decreased ratios of methanogen-to-bacterial 16S rRNA genes in all N_2_O-treated cultures (Fig. 3C–E), illustrating that N_2_O impacted the methanogen populations much more strongly than the bacterial populations. In the absence of N_2_O, the average growth yields of methanogens in the enrichment cultures with MeOH, H_2_, or acetate were 0.6 × 10^7^ ± 0.1 × 10^7^, 0.6 × 10^5^ ± 0.3 × 10^5^, and 1.0 × 10^6^ ± 0.1 × 10^6^ cells per μmol of CH_4_ formed, respectively. In the presence of 10 μM N_2_O, the growth yields of methanogens declined by ~90%, 60%, and 50% in enrichment cultures that received MeOH, H_2_, and acetate, respectively (Fig. 4B, D, and F). Only negligible CH_4_ production and methanogen growth were measured in all mixed cultures with 30 μM N_2_O (Fig. 4B, D, and F). Taken together, the results of the mixed culture studies corroborate that N_2_O concentrations in the low micromolar range exhibit pronounced inhibitory effects on hydrogenotrophic, methylotrophic, and acetoclastic methanogenesis in microbial communities.

### Kinetic studies confirm potent N_2_O inhibition of methane formation rates

Whole-cell suspension assays using *M. barkeri* and the methanogenic mixed cultures were performed to quantitatively assess the inhibitory effects of N_2_O on CH_4_ production from each methanogenic substrate (Table S1). The Michaelis–Menten single-substrate single-inhibitor model (*R*^2^ > 0.95) best explained the trends of CH_4_ production rates versus increasing substrate concentrations (Fig. 5). Among all assays with *M. barkeri* and the mixed cultures, maximum CH_4_ production rates (i.e. *V*_max_ values) of acetate-fed cultures were most strongly affected by increasing N_2_O concentrations, followed by the H_2_- and MeOH-fed cultures (Figs 2 and 3).

In the absence of N_2_O, the *V*_max_ values for CH_4_ production in MeOH-, H_2_-, or acetate-amended *M. barkeri* cell suspension assays were 440.4 ± 10.2, 138.1 ± 6.9, and 40.8 ± 4.7 nmol CH_4_ min^−1^ mg protein^−1^, respectively (Fig. 5A, Table 1). The addition of N_2_O decreased the *V*_max_ of CH_4_ production in MeOH-, H_2_-, and acetate-fed *M. barkeri* cell suspension assays to different extents. Rate data determined in *M. barkeri* suspensions assays with MeOH fit the Michaelis–Menten inhibition model best. The *V*_max_ values declined by ~45% and 57% to 244.1 ± 38.4 and 188.8 ± 32.5 nmol CH_4_ min^−1^ mg protein^−1^, respectively, in the presence of 100 and 200 μM N_2_O. The determined inhibitory constant, *K_I_*, of N_2_O on methylotrophic CH_4_ production was 130.9 ± 4.7 μM in *M. barkeri* cell suspensions (Table 1), indicating that N_2_O concentrations around 130 μM reduced the maximum CH_4_ production rate (*V*_max_) by 50%. More pronounced N_2_O inhibition was observed in *M. barkeri* whole-cell suspensions assays using H_2_ as the electron donor for CO_2_ reduction (Fig. 5B). The addition of 50 and 100 μM N_2_O reduced the *V*_max_ values in H_2_-fed *M. barkeri* cell suspension assays by ~40% and 57% to 82.8 ± 29.5 and 59.9 ± 5.9 nmol of CH_4_ min^−1^ mg protein^−1^, respectively. The model simulation determined a *K_I_* value of 90.6 ± 10.8 μM N_2_O (Fig. 5B, Table 1), indicating a stronger inhibition of N_2_O on CH_4_ production in H_2_- versus MeOH-amended *M. barkeri* cell suspension assays. The *M. barkeri* assays with acetate as substrate showed the most pronounced inhibition by N_2_O (Fig. 5C, Table 1). In assays amended with 20 and 40 μM N_2_O, the *V*_max_ values decreased by 21% and 44% to 32.1 ± 4.2 and 22.8 ± 2.5 nmol of CH_4_ min^−1^ mg protein^−1^, respectively. From the best-fit inhibition model, a *K_I_* value of 24.8 ± 2.6 μM was determined for N_2_O inhibition of acetoclastic methanogenesis in *M. barkeri* cell suspensions. Collectively, the cell suspension assays corroborate strong inhibitory effects of N_2_O on methanogenesis, with the kinetics of acetoclastic methanogenesis being most impacted by N_2_O.

Similar kinetic responses were observed for whole-cell suspension assays conducted with the methanogenic mixed cultures pregrown with the respective substrates (Fig. 5D–F). In the absence of N_2_O, the determined *V*_max_ values for mixed culture cell suspension assays that received MeOH, H_2_, or acetate were 209.0 ± 4.4, 105.8 ± 3.7, and 22.8 ± 0.7 nmol CH_4_ min^−1^ mg protein^−1^, respectively (Table 1). Notably, the methanogenic mixed cultures were more sensitive to N_2_O than axenic *M. barkeri* cultures irrespective of the type of methanogenic substrate provided. In cell suspension assays that received MeOH, the presence of 50 and 100 μM N_2_O reduced the *V*_max_ values by 37.4 ± 10.8% and 75.4 ± 21.4%, respectively, compared to assays without N_2_O. The inhibition model determined a *K_I_* value of 109.9 ± 6.8 μM for N_2_O inhibition on CH_4_ production in cell suspension assays amended with MeOH (Fig. 5D, Table 1). In H_2_-amended cell suspension assays, the presence of 30 and 60 μM N_2_O decreased the *V*_max_ values by 32.3 ± 9.8 and 65.4 ± 12.9%, respectively, compared to assays without N_2_O (Fig. 5E, Table 1). The best-fit inhibition model determined a *K_I_* value for N_2_O inhibition of 62.1 ± 6.4 μM for CH_4_ production in assays that received H_2_ as electron donor. Consistent with the observations made with *M. barkeri*, the most pronounced N_2_O inhibition on CH_4_ production rates was observed in mixed culture cell suspension assays that received acetate as substrate (Fig. 5F). In the presence of 10 and 30 μM N_2_O, the *V*_max_ values in suspension assays with acetate decreased by 38 ± 7.8% and 74.9 ± 16.0%, respectively, compared to assays without N_2_O. Using the best-fit Michaelis–Menten inhibition model, a *K_I_* value of 17.7 ± 1.8 μM was determined for N_2_O inhibition of CH_4_ production in cell suspension assays with acetate (Table 1).

Taken together, the experimental data demonstrate that dissolved N_2_O concentrations in the low μM range (i.e. 20–100 μM) repress CH_4_ production and reduce growth yields of *M. barkeri* in axenic culture and of different methanogen guilds in methanogenic mixed cultures. Evaluation of the kinetic data determined in cell suspension assays revealed distinct N_2_O inhibition patterns for CH_4_ production from acetate, H_2_, and MeOH, with the rate of acetoclastic CH_4_ production being most sensitive to N_2_O inhibition. The *K_I_* values for N_2_O inhibition of CH_4_ production from key methanogenic substrates ranged between 18 and 130 μM, suggesting N_2_O can impact CH_4_ production and emissions in diverse ecosystems, including critical zone environments (e.g. thawing permafrost).

## Discussion

Microorganisms drive global C and N cycling and ultimately control CH_4_ and N_2_O production, consumption, and thus emissions to the atmosphere [10, 11, 13]. Predictive climate models must consider the responses of microbial CH_4_ and N_2_O production under environmental change scenarios [10, 57]. Quantitative assessment of feedbacks that affect CH_4_ production are crucial for refining greenhouse gas emission models. This study investigated the feedback effects between two important greenhouse gases, specifically the inhibitory effect of N_2_O on archaeal CH_4_ production, to provide quantitative data that link environmental N_2_O concentrations with methanogenesis. The findings demonstrate that environmentally relevant, micromolar levels of N_2_O suppress CH_4_ production and the growth of methanogens. The determined *K_I_* values reveal a concentration-dependent, progressively negative feedback by N_2_O on archaeal CH_4_ formation rates and the total amounts of CH_4_ produced and show that the strength of the inhibition is most pronounced for acetoclastic methanogenesis.

Assuming the current day partial pressure of 335 ppb N_2_O in the atmosphere, the theoretical concentration of N_2_O in air-equilibrated water should be around 7 nM; however, substantially elevated levels of dissolved N_2_O have been observed in groundwater and watersheds [33, 42]. In areas impacted by agricultural activities (e.g. fertilizer application), dissolved groundwater N_2_O concentrations can exceed 100 μM [42]. Even in some remote, natural aquatic systems, such as ice-covered Antarctic lakes, N_2_O concentrations of up to 86 μM have been reported [58, 59]. N_2_O is generated during N cycling and major formation processes include microbial denitrification, ammonia oxidation, and abiotic chemodenitrification [44]. Based on the physiology of the microorganisms (e.g. ammonia oxidizers are strict aerobes) and the thermodynamics of the processes (e.g. nitrate and nitrite reduction are associated with a greater change in Gibbs free energy than methanogenesis), one might argue that N_2_O formation and methanogenesis are physically separate processes, thus limiting the exposure and inhibitory effects of N_2_O on methanogens. Such redox stratification does occur; however, most environmental matrices, such as soils, are highly heterogenous and characterized by dynamic spatial and temporal gradients resulting in patchy distribution of redox processes. N_2_O is water-soluble and, depending on hydrology, can reach other redox zones [60]. Consequently, impacts of elevated N_2_O on various biogeochemical processes, including those associated with greenhouse gas emissions, are likely. Laboratory studies have reported inhibitory effects of nitrogen oxides (NO*x*), including N_2_O, on methanogenesis [34–37, 61, 62]; however, the available data are scarce and no uniform pattern has emerged that would support a quantitative relationship between N_2_O and microbial CH_4_ production. Consequently, this negative feedback of N_2_O on CH_4_ production has not been considered in greenhouse gas emission models.

The evaluation of N_2_O inhibition on CH_4_ production from methanogenic substrates (i.e. MeOH, acetate, and H_2_/CO_2_) with both the model methanogen *M. barkeri* and digester sludge-derived mixed methanogenic cultures quantitatively links N_2_O concentrations with methanogen activity and growth. The growth experiments illustrate that micromolar N_2_O concentrations affect CH_4_ production, and the whole-cell suspension assays and kinetic model simulations provide a plausible explanation for the inconsistent literature reports about N_2_O effects on methanogenesis. Specifically, acetoclastic methanogenesis was most sensitive to N_2_O (*K_I_* values of 18–25 μM), followed by hydrogenotrophic (*K_I_* 60–90 μM) and methylotrophic methanogenesis (*K_I_* 110–130 μM), indicating that the type of methanogenic substrate utilized affects the sensitivity of methanogens to N_2_O. N_2_O inhibition was significantly more pronounced in methanogenic enrichment cultures than in axenic *M. barkeri* cultures, and 30 μM N_2_O prevented CH_4_ production and methanogen growth in the mixed culture experiments regardless of the type of methanogenic substrate utilized. Previous studies support that mixed methanogenic communities are more sensitive to N_2_O than commonly studied model methanogen isolates [34, 35, 62, 63]. These observations suggest that the axenic methanogen cultures used to elucidate the biochemistry and genetics of methanogenesis may not serve as general models for other features of methanogen biology (e.g. N_2_O inhibition). The reasons for the reduced sensitivity of axenic versus mixed methanogen cultures to N_2_O may be a result of long-term adaptation of the isolates to laboratory cultivation, or not-yet-characterized microbe–microbe interaction networks render mixed cultures more susceptible to N_2_O inhibition. The enrichment cultures used to determine the *K_I_* values for N_2_O inhibition harbored diverse methanogen groups (Fig. 3A), and kinetic studies (e.g. determination of *K_I_* values for N_2_O inhibition) with representative isolates of the various lineages are warranted to capture the breadth of methanogen responses to N_2_O.

Taken together, the low μM range *K_I_* values of N_2_O that impact methanogenesis suggest major consequences of rising N_2_O concentrations for C cycling. Of note, the vast majority of the annual ~1 billion metric tons of biogenic CH_4_ is generated from acetate- and H_2_-driven CO_2_ reduction [16–20, 64], the two processes with the lowest observed *K_I_* values for N_2_O inhibition. It is therefore reasonable to predict that elevated environmental N_2_O will impact CH_4_ production and methanogen growth in ecosystems with high bioavailable C and N loads, such as wetlands, sediments, and permafrost soils.

Key enzyme systems involved in CH_4_ production and energy conservation require cobamide prosthetic groups [65] (Fig. 1). The super-reduced Co(I) form of cobamides is susceptible to oxidants such as N_2_O, a plausible mechanism for the observed inhibition of methanogenesis [29, 34, 63]. The experimental efforts demonstrated that acetoclastic methanogenesis was most sensitive to N_2_O, with *K_I_* values in the range of 18–25 μM, indicating that N_2_O concentrations in the range of 20 μM would reduce the *V*_max_ of CH_4_ production from acetate by 50%. The *K_I_* values for N_2_O inhibition determined for hydrogenotrophic and methylotrophic methanogenesis ranged between 60–90 and 110–130 μM, respectively. The reasons for the apparently substrate-specific *K_I_* values for N_2_O inhibition likely reflect differences in the pathways leading to CH_4_ production from acetate, H_2_/CO_2_ and MeOH (Fig. 1). The acetoclastic pathway involves two steps catalyzed by corrinoid-dependent enzyme systems, CODH/ACS and the MTR corrinoid enzyme complex [16, 65]. (Fig. 1), both of which are targets for N_2_O inhibition. Distinct cobamide-dependent enzyme systems catalyze the formation of CH_3_–CoM in the hydrogenotrophic and the methylotrophic pathways [22, 66]. Both the acetoclastic and hydrogenotrophic pathways depend on the MTR corrinoid enzyme complex to generate CH_3_–CoM and to conserve energy [22, 27]. By contrast, methylotrophic methanogens can directly generate CH_3_–CoM without the energy-conserving MTR corrinoid enzyme complex from methylated compounds (e.g. MeOH) via a substrate-specific, corrinoid-dependent methyltransferase complex (i.e. MtaA, MtaB, and MtaC in the case of MeOH) [67]. The experimental efforts consistently demonstrated that CH_4_ production from acetate and H_2_/CO_2_ exhibited 7- and 3-fold higher sensitivities, respectively, to N_2_O than CH_4_ production from MeOH. These findings suggest that N_2_O inhibition of methanogenesis is related to the oxidation of the super-reduced Co(I) cobamide, which is essential for the corrinoid enzyme complexes involved in the different methanogenesis pathways. While the differential susceptibilities of pathway-specific corrinoid-dependent enzymatic steps can explain the distinct inhibitory effects of N_2_O on CH_4_ production via the acetoclastic, hydrogenotrophic, and methylotrophic pathways, detailed enzymatic studies would be needed to assess the responses of individual enzyme systems to N_2_O. Kinetic data for the enzymatic regeneration of the super-reduced Co(I) state are lacking, and the rates of reduction may differ between enzymes and pathways, which could contribute to the observed physiological response of decreased CH_4_ production. Recovery from N_2_O inhibition was not a focus, but the experimental data indicate partial recovery of methane formation in N_2_O-treated axenic and mixed cultures; however, the methanogen growth yields remained lower in N_2_O-treated cultures compared to controls without N_2_O even over the extended incubation period. Further studies are needed to generate mechanistic insights into modes of recovery from N_2_O inhibition.

The presence of N_2_O significantly decreased growth yields or completely abolished growth in the examined methanogenic cultures (Table 2). The type of methanogenic substrate utilized determines the fraction of electrons available from electron donor oxidation directed toward cell synthesis and thus governs the growth yields of methanogens [53, 68]. N_2_O affects corrinoid-dependent enzymes involved in electron transfer (e.g. the MTR enzyme complex), and it is not surprising that N_2_O interferes with energy conservation in methanogens. Consistently, enumeration of methanogen 16S rRNA genes at the termination of all growth experiments illustrated that N_2_O not only negatively impacted CH_4_ production but also methanogen growth yields. An alternate explanation for the reduced methanogen growth yields in the mixed cultures exposed to N_2_O could be competition for electron donor (e.g. H_2_); however, the sequencing and the qPCR data do not support this hypothesis, and N_2_O inhibition explains the decline of methanogens and the changes of methanogen-to-bacteria ratios. The measured methanogen growth yield data in the absence of N_2_O were on par with reported experimental data and closely matched the theoretical values (i.e. yields calculated based on thermodynamics) (Table 2). One exception were the growth yields measured in H_2_/CO_2_-fed *M. barkeri* cultures, which were ~10-fold lower than data reported in the literature. The most pronounced growth suppression was observed in the mixed methanogenic cultures, where 30 μM N_2_O was sufficient to prevent the growth of methanogenic archaea. Collectively, the data show that micromolar concentrations of N_2_O decrease or abolish CH_4_ production, reduce methanogen growth yields, and exhibit progressively negative feedback on microbial CH_4_ production.

Microbial processes are strongly influenced by environmental factors and their responses to climate change vary both spatially and temporally [11, 69]. To improve the predictive power of climate models and potentially justify the application of biotechnological approaches for managing greenhouse gas emissions, interactions and feedbacks between relevant biotic/abiotic processes must be understood and quantitatively captured [70]. Attempts have been made to include microbial data (i.e. biomass, enzyme, and growth kinetics) to improve climate models [70], but the incorporation of multifactorial, multidirectional, and often nonlinear biotic/abiotic feedbacks underlying the global CH_4_ and N_2_O budgets is challenging and requires robust quantitative data. The determined *K_I_* values for N_2_O inhibition of methanogenesis reveal a relevant negative feedback effect on CH_4_ emissions, and the new quantitative information generates opportunities to refine CH_4_ emission models.

## Supplementary Material

Yin_N2O_CH4_SI_10_wrae027

Supplemental_Table_S13_wrae027

## Data Availability

All data generated or analyzed during this study are included in this published article and its supplementary information files. The raw amplicon library reads are available in the SRA repository.
