# Supplementary material for: Nitrous oxide inhibition of methanogenesis represents an underappreciated greenhouse gas emission feedback
Source: ISME J. 2024 Mar 6;18(1):wrae027. doi: 10.1093/ismejo/wrae027 (PMC10960958; doi:10.1093/ismejo/wrae027)
Supplement: Yin_N2O_CH4_SI_10_wrae027 [file yin_n2o_ch4_si_10_wrae027.pdf]

Supplemental Information

Nitrous oxide inhibition of methanogenesis represents an underappreciated greenhouse gas emission feedback

Yongchao Yin[1,2,6 #], Fadime Kara-Murdoch[1,6 &], Robert W. Murdoch[2 &], Jun Yan[1,2,5], Gao Chen[1,3], Yongchao Xie[1,3 ‡], Yanchen Sun[1,3] and Frank E. Löffler[1,2,3,4,6 *]

[1]Center for Environmental Biotechnology, [2]Department of Microbiology, [3]Department of Civil and Environmental Engineering, [4]Department of Biosystems Engineering and Soil Science, University of Tennessee, Knoxville, Tennessee 37996, USA. [5]Key Laboratory of Pollution Control and Environmental Engineering, Institute of Applied Ecology, Chinese Academy of Sciences, Shenyang, Liaoning 110016, China. [6]Biosciences Division, Oak Ridge National Laboratory, Oak Ridge, Tennessee 37831, USA

Current addresses:

[#] Department of Biology, Northeastern University, Boston, MA 02148, USA

[&] Battelle Memorial Institute, Columbus, OH 43201, USA

[‡] Department of Chemistry and Biochemistry, University of California, Los Angeles, CA 90095, USA.

*Corresponding author: Frank E. Löffler, E-mail: frank.loeffler@utk.edu

Supplemental information summary:

Supplemental methods, 13 tables and references. Table S13 is in a separate file.

Supplemental Methods

Methanogenic cultures and growth condition

To determine if *M. barkeri* employing different methanogenic pathways exhibits different sensitivities to N_2O, cultures were pre-grown with MeOH, H_2/CO_2 or acetate for at least three consecutive transfers and then examined for the impact of N_2O on CH_4 production. Also analyzed were three methanogenic mixed cultures derived from an anaerobic digester (Kuwahee Wastewater Treatment Plant, Knoxville, TN, USA). The three different enrichments were derived from the same inoculum and enriched with H_2/CO_2, acetate, or MeOH for at least six consecutive transfers (inoculum size 3%, v/v).

N_2O stocks and calculation of aqueous phase N_2O concentrations. To achieve final aqueous N_2O concentrations ranging from 10 to 200 µM in cell suspension assay vials, 10-fold diluted (with N_2) N_2O gas stocks were prepared in sealed 60 mL serum bottles. Briefly, 60-mL serum bottles containing 3-5 glass beads (5 mm diameter, Sigma-Aldrich, St Louis, MO, USA) and sealed with thick butyl rubber stoppers were flushed with pure N_2 gas for 5 minutes before 6 mL of N_2 gas was replaced with 6 mL N_2O (⩾99%, Sigma-Aldrich, St Louis, MO, USA) using plastic syringes. After mixing the gas stock by manual shaking, different volumes of N_2O stock, 0.1 – 1.0 mL of N_2O gas (undiluted or 10-fold diluted in N_2), were withdrawn with plastic syringes equipped with 0.2 µm membrane filter units (Millipore, Sigma-Aldrich, St Louis, MO, USA) with 18-gauge needles and directly injected into the experimental vessels.

The volume of N_2O stock required in assay vials and final aqueous N_2O concentrations in culture vessels was calculated from the headspace concentration using a dimensionless Henry's constant for N_2O at 30°C of 1.94 based on the equation

$C_{aq} = C_g/H_{cc}$ [1, 2]. C_{aq} is the aqueous N_2O concentration (μM), C_g is the headspace N_2O concentration (μmol L^{-1}), and H_{cc} is the Henry's constant (dimensionless). For example, to achieve 100 μM aqueous phase N_2O in 160 mL vessels with 100 mL of medium and a 60 mL headspace, a total 523 μL of pure N_2O gas was directly injected into each vessel. Based on Henry's law using a dimensionless Henry's constant of 1.94 for N_2O, a volume of 282 μL N_2O existed in the 60 mL headspace and 241 μL N_2O dissolves in 100 mL of the medium resulting in an aqueous phase concentration of 100 μM after equilibration. After equilibration, the headspace N_2O concentrations in the assay vials were measured by injecting 100 μL headspace samples into an Agilent 7890A GC equipped with an HP-PLOT Q column (30 m length × 0.320 mm diameter, 20 μm film thickness) and a microelectron capture detector as previously described [3].

Whole cell suspension assays. The substrate concentration ranges (**Tables S2**) used in cell suspension assays were chosen so that both unsaturated and saturated states of active cells were captured and K_m and V_{max} values within the chosen initial substrate concentrations could be captured [4, 5]. Specifically, cell suspension assays were performed in 20-mL glass vials flushed with N_2/CO_2 (80/20, v/v) and sealed with Teflon-lined butyl rubber stoppers held in place with aluminum crimps. A series of 8 to 10 assay vials, each with a fixed initial substrate concentration [S] as listed in **Tables S3-S8**, were prepared for each substrate assay (i.e., MeOH, acetate, and H_2). After equilibration, assay vials received 0.1 mL of 1,000-fold concentrated cell suspensions to initiate activity. That is, each assay vial received a fixed initial substrate concentration [S] and the same abundance of active cells (i.e., cell titers), and the amount of CH_4 produced was measured six times over a 3-hour incubation period. The corresponding

initial CH_4 production rates associated with each substrate concentration [S] were then determined from the slopes of the progression curves of CH_4 production over time. Note that the linear regression analysis (i.e., the slope) of the progression curves of CH_4 produced over time in each assay vial included at least four measurements. Using such a monitoring scheme, one initial CH_4 production rate V was generated from one fixed initial substrate concentration [S]. The initial CH_4 production rate V increases with initial substrate concentration [S] until reaching saturation with maximum CH_4 production rates. Preliminary experiments determined the appropriate cell titers and substrate concentrations so that no more than 50% of initial substrate had been consumed at the end of the 3-hour incubation period and the initial CH_4 production rates were not impacted by acetate, H_2, or MeOH availability.

The resulting initial CH_4 production rates V determined with different initial substrate concentrations [S] were then used as input values for Michaelis-Menten one-substrate one-inhibitor models to determine the kinetic parameters. The maximum CH_4 production rate V_{max} and the half-velocity constant K_m for each treatment were calculated using the Michaelis-Menten nonlinear regression models using *R*. Data sets from assays amended with increasing N_2O concentrations were fit to the competitive, non-competitive, and un-competitive inhibition models to determine the inhibitory constant, K_I, of N_2O on CH_4 production from the different substrates. The best-fit inhibition model presented was chosen based on the highest coefficient of determination (R^2) and the lowest standard deviation of the residuals (Sy.x.). From the best-fit inhibition models, the Michaelis-Menten plots were generated for each

methanogenic substrate (i.e., acetate, MeOH, and H_2) for data visualization and the determination of K_I.

16S rRNA gene amplicon sequencing. 16S rRNA genes were amplified using the 341F/785R primer pair (CCTACGGGNGGCWGCAG / GACTACHVGGGTATCTAATCC) [6]. The resulting PCR amplicons were purified and sequenced on an Illumina MiSeq in 275 base pair (bp) paired end mode. The amplicon sequencing data were analyzed using a custom workflow constructed in the QIIME 2 environment [7]. Adapters were removed using Cutadapt [8]. Reads were then trimmed, error corrected, and purged of chimeric amplicons using dada2 operated in paired-end mode with no read truncation [9]. The resulting actual sequence variants (ASVs) were classified using the QIIME 2 sklearn feature classifier against a pretrained Silva v138 99% taxonomic classifier [10].

Archaeal reference tree generation. Available archaeal 16S rRNA gene sequences of at least 1,300 bp in length were downloaded from the Integrated Microbial Genomes database [11] (806 sequences) and subjected to 97% identity clustering using CD-HIT [12]. The resulting 297 representative sequences were aligned with MAFFT-auto [13]. The alignment was trimmed using TrimAl with -gappyout [14] and subjected to maximum likelihood phylogenetic reconstruction using RaxML v8.2.12 [15] with rapid bootstrapping, GTR gamma likelihood substitution matrix, and autoMRE bootstopping. RaxML was operated via the CIPRES Science Gateway [16]. ASV sequences classified as archaeal were added to the archaeal reference tree using MAFFT –add fragments and RaxML evolutionary placement algorithm (EPA, -f v). The resulting *.jplace file was visualized at the Interactive Tree of Life website [17].

Table S1. Whole cell suspension assays to determine CH_4 production kinetics.

Culture	Substrate and Inhibitor Concentrations (μM)					
	MeOH	N_2O	H_2	N_2O	Acetate	N_2O
M. barkeri	200 – 7 500	0, 100, 200	1.0 – 333	0, 50, 100	2 500 – 50 000	0, 20, 40
Mixed cultures	100 – 10 000	0, 50, 100	0.2 – 333	0, 30, 60	2.5 – 20 000	0, 10, 30

Table S2. Initial CH_4 production rates versus acetate concentrations in *M. bakeri* cell suspension assays in the presence of 0, 20 and 40 μM N_2O.

Acetate (μM)	*V* [a]	Acetate (μM)	*V* [a]	Acetate (μM)	*V* [a]
No N_2O		20 μM N_2O		40 μM N_2O	
2.5	0.44	2.5	0.33	2.5	0.10
5.0	9.70	5.0	7.36	5.0	3.24
7.5	12.4	7.5	9.22	7.5	3.92
10.0	17.8	10.0	12.51	10.0	5.20
15.0	25.8	15.0	13.18	15.0	8.35
20.0	27.3	20.0	15.07	20.0	9.14
30.0	29.5	30.0	16.41	30.0	8.44
40.0	28.4	40.0	18.55	40.0	10.47
50.0	29.2	50.0	19.22	50.0	9.94

[a] Initial CH_4 production rate (nmol CH_4 produced min^{-1} mg $protein^{-1}$)

Table S3. Initial CH_4 production rates versus H_2 concentrations in *M. barkeri* whole cell suspension assays in the presence of 0, 50 and 100 μM N_2O.

**H_2 (μM)**	***V*** [a]	**H_2 (μM)**	***V*** [a]	**H_2 (μM)**	***V*** [a]
No N_2O		50 μM N_2O		100 μM N_2O	
1.1	7.91	1.1	3.30	1.1	0.91
2.1	17.44	2.1	5.49	2.1	1.62
4.2	24.40	4.2	9.79	4.2	2.92
8.4	30.18	8.4	13.90	8.4	6.19
21.0	47.56	21.0	23.89	21.0	13.01
41.6	53.22	41.6	36.22	41.6	22.56
83.3	85.35	83.3	56.59	83.3	38.01
208.0	103.46	208.0	80.67	208.0	48.91
333.0	122.12	333.0	84.55	333.0	54.88

[a] Initial CH_4 production rate (nmol CH_4 produced min^{-1} mg $protein^{-1}$)

Table S4. Initial CH_4 production rates versus MeOH concentrations in *M. barkeri* whole cell suspension assays in the presence of 0, 100 and 200 μM N_2O.

**MeOH (μM)**	***V*** [a]	**MeOH (μM)**	***V*** [a]	**MeOH (μM)**	***V*** [a]
No N_2O		100 μM N_2O		200 μM N_2O	
125	25.1	125	15.9	100	8.9
250	47.8	250	28.2	250	21.4
500	80.8	500	52.3	500	37.0
750	106.2	750	75.5	1000	69.1
1250	163.8	1250	101.1	2000	96.0
2000	220.0	2000	124.3	3750	119.0
2500	253.7	2500	159.1	5000	128.7
3700	296.8	3700	181.6	7500	131.4
5000	328.2	5000	187.0		

7500	352.8	7500	200.0		

[a] Initial CH_4 production rate (nmol CH_4 produced min^{-1} mg $protein^{-1}$)

Table S5. Initial CH_4 production rates versus acetate concentrations in acetate-enriched mixed culture whole cell suspension assays in the presence of 0, 10 and 30 μM N_2O.

**Acetate (μM)**	***V*** [a]	**Acetate (μM)**	***V*** [a]	**Acetate (μM)**	***V*** [a]
No N_2O		10 μM N_2O		30 μM N_2O	
2.5	0.72	2.5	0.22	2.5	0.22
5.0	1.64	5.0	0.62	5.0	0.29
10	2.91	10	1.51	10	0.35
100	5.91	50	2.88	50	0.92
500	12.84	100	3.27	100	2.64
1 000	18.12	1 000	12.41	1 000	5.97
5 000	20.38	5 000	13.95	5 000	6.04
10 000	21.12	10 000	15.22	10 000	6.17
20 000	23.54	20 000	17.04	20 000	7.69

[a] Initial CH_4 production rate (nmol CH_4 produced min^{-1} mg $protein^{-1}$)

Table S6. Initial CH_4 production rates versus H_2 concentrations in H_2/CO_2-enriched mixed culture whole cell suspension assays in the presence of 0, 30 and 60 μM N_2O.

**H_2 (μM)**	***V*** [a]	**H_2 (μM)**	***V*** [a]	**H_2 (μM)**	***V*** [a]
No N_2O		30 μM N_2O		60 μM N_2O	
0.2	0.5	0.2	0.3	2.1	0.2
2.1	4.9	2.1	2.7	4.2	3.7
4.2	11.3	4.2	8.6	8.4	10.2
8.4	24.6	8.4	17.5	21.0	29.8
21.0	66.4	21.0	45.3	41.6	39.8
41.6	79.6	41.6	50.8	83.3	45.4
83.3	88.4	83.3	58.7	165.5	50.7
166.5	90.3	208	60.6	333	52.4
333.0	97.1	249.6	63.2		

		333.0	66.6		

[a] Initial CH_4 production rate (nmol CH_4 produced min^{-1} mg $protein^{-1}$)

Table S7. Initial CH_4 production rates versus MeOH concentrations in MeOH-enriched mixed culture whole cell suspension assays in the presence of 0, 50 and 100 µM N_2O.

**MeOH (µM)**	***V*** [a]	**MeOH (µM)**	***V*** [a]	**MeOH (µM)**	***V*** [a]
No N_2O		50 µM N_2O		100 µM N_2O	
2.5	1.4	2.5	0.9	2.5	0.6
10	5.6	10	3.6	10	2.7
25	13.4	25	7.6	25	6.7
100	43.8	100	25.5	100	25.1
500	124.9	500	78.6	500	57.5
1 000	158.5	1000	115.1	1 000	78.6
2 000	169.8	2 000	137.6	2 000	86.6
3 500	191.5	3 500	138.8	3 500	90.4
6 000	190.5	6 000	142	6 000	96.4

[a] Initial CH_4 production rate (nmol CH_4 produced min^{-1} mg $protein^{-1}$)

Table S8. Inhibition models used in whole cell suspension assays.

Michaelis-Menten Equation: $$v_0 = \frac{V_{\max}\,[S]}{K_m + [S]}$$	Uncompetitive inhibition: $v_0 = \frac{V_{\max}\,[S]}{(K_m + \alpha[S])}$ (3)
Competitive inhibition: $v_0 = \frac{V_{\max}\,[S]}{(\alpha K_m + [S])}$ (1)	Noncompetitive inhibition: $v_0 = \frac{V_{\max}\,[S]}{\alpha(K_m + [S])}$ (2)
For simplification, the factor of inhibitor concentrations and inhibition constants in equations (1) to (3) are shown as α, whereby $\alpha = 1 + \frac{[I]}{K_i}$	

Table S9. Statistical parameters (R^2 and Sy.x values) used for determining the best-fit inhibition models and inhibition constants in cell suspension amended with N_2O as inhibitor.

Culture	Substrate	Inhibitor	Tested models	Statistical Parameters		K_I (μM)
				R^2	Sy.x	
			**Noncompetitive**	**0.97**	**2.41**	**24.8 ± 3.1**
M. barkeri	Acetate	N_2O	Uncompetitive	0.96	2.77	14.9 ± 2.6
			Competitive	0.95	2.99	7.7 ± 1.8
			**Noncompetitive**	**0.98**	**6.36**	**90.6 ± 10.8**
M. barkeri	H_2	N_2O	Competitive	0.98	7.55	16.6 ± 3.6
			Uncompetitive	0.97	8.26	66.0 ± 11.5
			Noncompetitive	0.99	10.11	80.1 ± 5.3
M. barkeri	MeOH	N_2O	**Uncompetitive**	**0.99**	**7.03**	**130.9 ± 4.7**
			Competitive	-	-	-
			**Uncompetitive**	**0.99**	**1.17**	**17.7 ± 1.8**
Mixed culture	Acetate	N_2O	Noncompetitive	0.99	10.11	15.9 ± 1.8
			Competitive	0.95	2.71	0.6 ± 0.3
			Uncompetitive	0.98	6.34	50.9 ± 6.9
Mixed culture	H_2	N_2O	**Noncompetitive**	**0.99**	**5.15**	**62.1 ± 6.4**
			Competitive	0.95	9.88	11.3 ± 4.3
			**Noncompetitive**	**0.99**	**5.99**	**109.9 ± 6.8**
Mixed culture	MeOH	N_2O	Uncompetitive	0.99	7.92	91.0 ± 7.9
			Competitive	0.97	15.94	13.7 ± 4.6

R^2, the Coefficient of Determination, gives information about the fit of the measured data to the different models tested, and the model with the highest R^2 value provides the best data fit.

The Sy.x represents the Standard Deviation of the Residuals, and the model with the lowest Sy.x value provides the best prediction of the data.

In all cell suspensions assays, the best fit inhibition Models (highlighted in bold) gave the highest R^2 and lowest Sy.x values, and the kinetic parameters extracted from the best fit models are included in the main text.

Table S10. Primers and probes used in this study [18].

Target DNA	Primer and Probe sets	Sequence (5' – 3')
Total Bacteria 16S rRNA gene	Bac1055YF	ATGGYTGTCGTCAGCT
	Bac1392R	ACGGGCGGTGTGTAC
	Bac1115-probe	FAM-CAACGAGCGCAACCC-MGB
Total Archaea 16S rRNA gene	Mtgen835F	GGGRAGTACGKYCGCAAG
	Mtgen918R	GAVTCCAATTRARCCGCA
	Mtgen831-probe	FAM-CCAATTCCTTTAAGTTTCA-MGB

Table S11. Growth yield calculations based on qPCR enumeration of archaeal 16S rRNA genes in axenic *M. barkeri* and methanogenic mixed cultures.

Culture	Substrate	N_2O (μM)	Methanogen 16S rRNA gene copies mL^{-1}		CH_4 yield (μmol)	Methanogen Yield [a] (16S rRNA gene copies μmol CH_4^{-1})
			Inoculum	Final		
M. barkeri	Acetate	0	$1.68(\pm0.21) \times 10^6$	$3.73(\pm1.06) \times 10^9$	580.6 ± 7.4	$6.43(\pm2.03) \times 10^6$
	Acetate	20	$1.68(\pm0.21) \times 10^6$	$6.05(\pm1.20) \times 10^7$	20.2 ± 1.7	$4.52(\pm0.71) \times 10^6$
	Acetate	50	$1.68(\pm0.21) \times 10^6$	$9.24(\pm1.10) \times 10^7$	19.1 ± 1.4	$3.13(\pm0.91) \times 10^6$
M. barkeri	H_2	0	$1.68(\pm0.21) \times 10^6$	$7.54(\pm1.91) \times 10^8$	317.0 ± 2.8	$2.37(\pm0.62) \times 10^6$
	H_2	50	$1.68(\pm0.21) \times 10^6$	$2.15(\pm0.19) \times 10^8$	315.5 ± 3.0	$0.68(\pm0.07) \times 10^6$
	H_2	100	$1.68(\pm0.21) \times 10^6$	$0.13(\pm0.04) \times 10^8$	33.4 ± 5.8	$0.37(\pm0.09) \times 10^6$
M. barkeri	MeOH	0	$1.68(\pm0.21) \times 10^6$	$10.10(\pm0.85) \times 10^9$	635.8 ± 1.2	$1.59(\pm0.14) \times 10^7$
	MeOH	100	$1.68(\pm0.21) \times 10^6$	$3.64(\pm0.73) \times 10^9$	629.6 ± 5.0	$0.58(\pm0.13) \times 10^7$
	MeOH	200	$1.68(\pm0.21) \times 10^6$	$0.13(\pm0.06) \times 10^9$	48.6 ± 5.2	$0.27(\pm0.14) \times 10^7$
Mixed	Acetate	0	$1.02(\pm0.03) \times 10^6$	$1.44(\pm0.11) \times 10^9$	589.1 ± 23.6	$2.42(\pm0.18) \times 10^6$
	Acetate	10	$1.02(\pm0.03) \times 10^6$	$0.32(\pm0.19) \times 10^9$	267.9 ± 33.2	$1.15(\pm0.73) \times 10^6$
	Acetate	30	$1.02(\pm0.03) \times 10^6$	$0.09(\pm0.02) \times 10^9$	1.2 ± 1.3	-[b]
Mixed	H_2	0	$7.45(\pm0.77) \times 10^3$	$5.01(\pm2.33) \times 10^7$	316.0 ± 5.2	$1.59(\pm0.78) \times 10^5$
	H_2	10	$7.45(\pm0.77) \times 10^3$	$0.35(\pm0.32) \times 10^7$	67.9 ± 11.5	$0.60(\pm0.05) \times 10^5$
	H_2	30	$7.45(\pm0.77) \times 10^3$	$0.11(\pm0.00) \times 10^7$	0.03 ± 0.03	-[b]
Mixed	MeOH	0	$2.90(\pm0.00) \times 10^6$	$8.62(\pm1.02) \times 10^9$	541.5 ± 55.2	$1.59(\pm0.21) \times 10^7$
	MeOH	10	$2.90(\pm0.00) \times 10^6$	$0.12(\pm0.04) \times 10^9$	225.9 ± 22.6	$0.05(\pm0.02) \times 10^7$
	MeOH	30	$2.90(\pm0.00) \times 10^6$	$0.18(\pm0.08) \times 10^9$	2.4 ± 2.2	-[b]

[a] The *M. barkeri* genome has three 16S rRNA gene copies, and the qPCR numbers were divided by a factor of three to obtain the cell numbers. The calculation of methanogen cell numbers in the mixed cultures assumed an average 16S rRNA gene content of 2.5.

[b] All of the mixed cultures that received 30 μM N_2O produced negligible amount of CH_4 and growth was not apparent.

Table S12. Relative abundances of archaeal taxa (as percentage of total Archaea detected) in methanogenic enrichment cultures grown with different substrates and exposed/unexposed to N_2O. Values are derived from taxonomic classification of actual sequence variants (ASVs) generated by analyses of 16S rRNA gene amplicon sequencing data.

**Taxonomic Classification**			**H_2**		**Acetate**		**MeOH**	
**Order**	**Family**	**Genus**	**no N_2O**	**N_2O[a]**	**no N_2O**	**N_2O[a]**	**no N_2O**	**N_2O[a]**
Methanobacteriales	*Methanobacteriaceae*	unidentified	53.97%	55.06%	0.08%	0.00%	0.00%	0.00%
Methanobacteriales	*Methanobacteriaceae*	*Methanobacterium*	46.00%	44.91%	2.54%	1.79%	0.18%	0.45%
Methanomicrobiales	*Methanomicrobiaceae*	*Methanoculleus*	0.00%	0.00%	1.21%	0.23%	0.00%	0.00%
Methanomicrobiales	*Methanomicrobiaceae*	*Methanofollis*	0.00%	0.00%	0.08%	0.00%	0.00%	0.00%
Methanosarciniales	*Methanosaetaceae*	*Methanosaeta*	0.00%	0.00%	0.00%	0.00%	0.00%	0.32%
Methanosarciniales	*Methanosarcinaceae*	*Methanomethylovorans*	0.03%	0.00%	0.00%	0.00%	90.54%	84.20%
Methanosarciniales	*Methanosarcinaceae*	*Methanosarcina*	0.00%	0.00%	96.09%	97.98%	0.74%	0.95%
Methanomassiliicoccales	*Methanomassiliicoccaceae*	*Methanomassiliicoccus*	0.00%	0.02%	0.00%	0.00%	8.48%	14.08%
Methanomassiliicoccales	*Methanomassiliicoccaceae*	uncultured	0.00%	0.00%	0.00%	0.00%	0.07%	0.00%
Methanomassiliicoccales	*Methanomethylophilaceae*	*Ca.* Methanogranum	0.00%	0.01%	0.00%	0.00%	0.00%	0.00%

[a] Cultures was provided with 100 μM N_2O at the start of the sixth transfers.

References

1. Sander R. Compilation of Henry's law constants for inorganic and organic species of potential importance in environmental chemistry. Max-Planck Institute of Chemistry, Air Chemistry Department Mainz, Germany; 1999.
2. Sander R. Compilation of Henry's law constants (version 4.0) for water as solvent. Atmos Chem Physics. 2015;15(8):4399-4981.
3. Yin Y, Yan J, Chen G, Murdoch FK, Pfisterer N, Löffler FE. Nitrous oxide is a potent inhibitor of bacterial reductive dechlorination. Environ Sci Technol. 2018;53(2):692-701.
4. Fukagawa Y, Sakamoto M, Ishikura T. Micro-computer analysis of enzyme-catalyzed reactions by the Michaelis-Menten equation. Agri Biol Chem. 1985;49(3):835-837.
5. Michaelis L, Menten ML. Die Kinetik der Invertinwirkung. Biochem Zeitschrift. 1913;49:333-369.
6. Herlemann DPR, Labrenz M, Jürgens K, Bertilsson S, Waniek JJ, Andersson AF. Transitions in bacterial communities along the 2000 km salinity gradient of the Baltic Sea. The ISME J. 2011;5(10):1571-1579.
7. Bolyen E, Rideout JR, Dillon MR, Bokulich NA, Abnet CC, Al-Ghalith GA, et al. Reproducible, interactive, scalable and extensible microbiome data science using QIIME 2. Nature Biotechnol. 2019;37(8):852-857.
8. Martin M. Cutadapt removes adapter sequences from high-throughput sequencing reads. EMBnet J. 2011;17(1):10-12.
9. Callahan BJ, McMurdie PJ, Rosen MJ, Han AW, Johnson AJA, Holmes SP. DADA2: high-resolution sample inference from Illumina amplicon data. Nat Methods. 2016;13(7):581-583.
10. Quast C, Pruesse E, Yilmaz P, Gerken J, Schweer T, Yarza P, et al. The SILVA ribosomal RNA gene database project: improved data processing and web-based tools. Nucleic Acids Res. 2012;41(D1):D590-D596.
11. Chen I-MA, Chu K, Palaniappan K, Pillay M, Ratner A, Huang J, et al. IMG/M v.5.0: an integrated data management and comparative analysis system for microbial genomes and microbiomes. Nucleic Acids Res. 2018;47(D1):D666-D677.
12. Li W, Godzik A. Cd-hit: a fast program for clustering and comparing large sets of protein or nucleotide sequences. Bioinformatics. 2006;22(13):1658-1659.
13. Katoh K, Standley DM. MAFFT Multiple Sequence Alignment Software Version 7: Improvements in Performance and Usability. Mol Biol Evol. 2013;30(4):772-780.
14. Capella-Gutiérrez S, Silla-Martínez JM, Gabaldón T. trimAl: a tool for automated alignment trimming in large-scale phylogenetic analyses. Bioinformatics. 2009;25(15):1972-1973.
15. Stamatakis A. RAxML version 8: a tool for phylogenetic analysis and post-analysis of large phylogenies. Bioinformatics. 2014;30(9):1312-1313.
16. Miller MA, Pfeiffer W, Schwartz T, editors. Creating the CIPRES Science Gateway for inference of large phylogenetic trees. 2010 Gateway Computing Environments Workshop (GCE); 2010 14-14 Nov. 2010.
17. Letunic I, Bork P. Interactive tree of life (iTOL) v3: an online tool for the display and annotation of phylogenetic and other trees. Nucleic Acids Res. 2016;44(W1):W242-W245.
18. Kleindienst S, Chourey K, Chen G, Murdoch RW, Higgins SA, Iyer R, et al. Proteogenomics reveals novel reductive dehalogenases and methyltransferases expressed during anaerobic dichloromethane metabolism. Appl Environ Microbiol. 2019;85(6):e02768-02718.
