# Supplementary material for: Nitrous oxide inhibition of methanogenesis represents an underappreciated greenhouse gas emission feedback
Source: ISME J. 2024 Mar 6;18(1):wrae027. doi: 10.1093/ismejo/wrae027 (PMC10960958; doi:10.1093/ismejo/wrae027)
Supplement: Supplemental_Table_S13_wrae027 [file supplemental_table_s13_wrae027.pdf]

Table S13. Relative abundances of the taxa represented by the actual sequence variants derived from 16S rRNA gene amplicon library analysis of all mixed culture samples. Taxonomy is presented to genus-level resolution.

Relative abuandance of indicated taxon											
**Taxonomy**						**Sample name (treatment)**					
kingdom	phylum	class	order	family	genus	YCY-17 (H2)	YCY-18 (H2 + N2O)	YCY-19 (Acetate)	YCY-20 (Acetate + N2O)	YCY-21 (MeOH)	YCY-22 (MeOH + N2O)
d__Bacteria	p__Campilobacterota	c__Campylobacteria	o__Campylobacterales	f__Sulfurospirillaceae	g__Sulfurospirillum	0.005404085	0.274561404	0.000281326	0.000867364	0.024420045	0.607511258
d__Bacteria	p__Proteobacteria	c__Gammaproteobacteria	o__Enterobacterales	f__Enterobacteriaceae	g__Citrobacter	0	0	0.097863801	0.354306343	0.001322264	0.000266199
d__Bacteria	p__Bacteroidota	c__Bacteroidia	o__Sphingobacteriales	f__Lentimicrobiaceae	g__Lentimicrobium	0.001038073	0.005868839	0.222866145	0.015284355	0.00182439	0.000421482
d__Archaea	p__Halobacterota	c__Methanosarcinia	o__Methanosarciniales	f__Methanosarcinaceae	g__Methanomethylovorans	9.16E-05	0	0	0	0.222073444	0.041260898
d__Archaea	p__Euryarchaeota	c__Methanobacteria	o__Methanobacteriales	f__Methanobacteriaceae	__	0.160412787	0.193107769	0.00011253	0	0	0
d__Archaea	p__Euryarchaeota	c__Methanobacteria	o__Methanobacteriales	f__Methanobacteriaceae	g__Methanobacterium	0.1367203	0.157539683	0.003469682	0.002742745	0.000435176	0.000221833
d__Bacteria	p__Firmicutes	c__Clostridia	o__Peptostreptococcales-Tissierellales	f__Dethiosulfatibacteraceae	g__Dethiosulfatibacter	0.155069765	0.022911445	0	0	0	0
d__Archaea	p__Halobacterota	c__Methanosarcinia	o__Methanosarciniales	f__Methanosarcinaceae	g__Methanosarcina	0	0	0.131229018	0.150171129	0.001807652	0.000465849
d__Bacteria	p__Proteobacteria	c__Gammaproteobacteria	o__Burkholderiales	f__Rhodocyclaceae	g__uncultured	0.001343388	0.123830409	0.000262571	0.000328192	0.001138152	0.000532399
d__Bacteria	p__Bacteroidota	c__Bacteroidia	o__Bacteroidales	f__Williamwhitmaniaceae	g__Blvii28_wastewater-sludge_group	0	0.000146199	0.05570247	0.008532983	0.121564624	0.004347923
d__Bacteria	p__Firmicutes	c__uncultured	o__uncultured	f__uncultured	g__uncultured	0.116783195	0.012949039	0.015116563	0.007431197	0.005958558	0
d__Bacteria	p__Bacteroidota	c__Bacteroidia	o__Bacteroidales	f__Rikenellaceae	g__uncultured	0.112600372	0.04235589	0	9.38E-05	0	0
d__Bacteria	p__Spirochaetota	c__Spirochaetia	o__Spirochaetales	f__Spirochaetaceae	g__uncultured	0.000305316	0.000626566	0.066336578	0.069224999	0.107237305	0.063577275
d__Bacteria	p__Firmicutes	c__Clostridia	o__Clostridiales	f__Clostridiaceae	g__Clostridium_sensu_stricto_16	0.102311239	0.002694236	0	0	5.02E-05	0
d__Bacteria	p__Cloacimonadota	c__Cloacimonadia	o__Cloacimonadales	f__Cloacimonadaceae	g__Candidatus_Cloacimonas	0	0	0	0	0.099270244	0.057476874
d__Bacteria	p__Firmicutes	c__Clostridia	o__Eubacteriales	f__Eubacteriaceae	g__Acetobacterium	0.000854884	0.001086048	0.000300081	0	0.000251063	0.0001331
d__Bacteria	p__Bacteroidota	c__Bacteroidia	o__Bacteroidales	f__ML635J-40_aquatic_group	g__ML635J-40_aquatic_group	0	0	0.081978282	0.056495851	0.001472902	0
d__Bacteria	p__Synergistota	c__Synergistia	o__Synergistales	f__Synergistaceae	g__Syner-01	0.081213935	0.013011696	0.04624993	0.049134981	0	0
d__Bacteria	p__Desulfobacterota	c__Desulfovibrionia	o__Desulfovibrionales	f__Desulfovibrionaceae	g__Desulfovibrio	0.00314475	0.013784461	0.004407434	0.048525482	0.078432029	0.007675414
d__Bacteria	p__Bacteroidota	c__Bacteroidia	o__Bacteroidales	f__Paludibacteraceae	g__uncultured	0.077489085	0.001023392	0	0	0	0
d__Bacteria	p__Cloacimonadota	c__Cloacimonadia	o__Cloacimonadales	f__Cloacimonadaceae	g__LNR_A2-18	0	0	0	0	0	0
d__Bacteria	p__Chloroflexi	c__Anaerolineae	o__Anaerolineales	f__Anaerolineaceae	__	0	0	0	0.002391111	0.000719713	0.001242264
d__Bacteria	p__Cloacimonadota	c__Cloacimonadia	o__Cloacimonadales	f__Cloacimonadaceae	g__W5	0	0	0	0	0.06423861	0.047472215
d__Bacteria	p__Bacteroidota	c__Bacteroidia	o__Bacteroidales	f__Rikenellaceae	g__DMER64	0	0	0.000168795	7.03E-05	0	0
d__Bacteria	p__Bacteroidota	c__Bacteroidia	o__Bacteroidales	f__Dysgonomonadaceae	g__Petrimonas	0.000244252	0.000355054	0.062041674	0.014323222	0.00222609	0.002218328
d__Bacteria	p__Firmicutes	c__Clostridia	o__Clostridia	f__Hungateiclostridiaceae	g__uncultured	0	0	0.058384441	0.006001219	0	0
d__Bacteria	p__Proteobacteria	c__Gammaproteobacteria	o__Enterobacterales	f__Enterobacteriaceae	__	0	0	0.014910257	0.05462047	0.00020085	0
d__Bacteria	p__Armatimonadota	c__uncultured	o__uncultured	f__uncultured	g__uncultured	0	0	0	0	0.00169049	0.000798598
d__Bacteria	p__Synergistota	c__Synergistia	o__Synergistales	f__Synergistaceae	g__JGI-0000079-D21	0	0	0	0	0.053175108	0.01752479
d__Bacteria	p__Synergistota	c__Synergistia	o__Synergistales	f__Synergistaceae	g__uncultured	0	0	0	7.03E-05	0.000351488	0.000465849
d__Bacteria	p__Desulfobacterota	c__Desulfovibrionia	o__Desulfovibrionales	f__Desulfomicrobiaceae	g__Desulfomicrobium	0	0.000167084	0	0	0.010327051	0.04831518
d__Bacteria	p__Bacteroidota	c__Bacteroidia	o__Bacteroidales	f__Dysgonomonadaceae	g__Proteiniphilum	0	0.000856307	0.042949043	0.006938909	0.000318013	0.015949777
d__Bacteria	p__Bacteroidota	c__Bacteroidia	o__Bacteroidales	f__Rikenellaceae	g__U29-B03	0	0	0	0	0	0
d__Bacteria	p__Bacteroidota	c__Bacteroidia	o__Bacteroidales	f__Rikenellaceae	g__S50_wastewater-sludge_group	0	0	0	0	0.043919258	0.00270636
d__Bacteria	p__Synergistota	c__Synergistia	o__Synergistales	f__Synergistaceae	g__Thermovirga	0	0	0.012603387	0.042688359	0.000150638	0
d__Bacteria	p__Acidobacteriota	c__Holophagae	o__Holophagales	f__Holophagaceae	g__Holophagaceae	0	0	0	0	0	0
d__Bacteria	p__Firmicutes	c__Clostridia	o__Clostridiales	f__Clostridiaceae	g__Clostridium_sensu_stricto_7	0.009251061	0.040580618	0.001031527	0.001617516	0.009506913	0.001752479
d__Bacteria	p__Desulfobacterota	c__Desulfuromonadia	o__Geobacterales	f__Geobacteraceae	g__Trichlorobacter	0.000671694	0.03855472	0	0	0.000468651	0
d__Bacteria	p__Bacteroidota	c__Bacteroidia	o__Bacteroidales	f__Bacteroidetes_vadinHA17	g__Bacteroidetes_vadinHA17	0	0	0	0	0.000184113	0
d__Bacteria	p__Chloroflexi	c__Anaerolineae	o__Anaerolineales	f__Anaerolineaceae	g__uncultured	6.11E-05	0	0	0	0	0
d__Bacteria	p__Firmicutes	c__Syntrophomonadia	o__Syntrophomonadales	f__Syntrophomonadaceae	__	0	0	0.000300081	7.03E-05	0	0
d__Bacteria	p__Cloacimonadota	c__Cloacimonadia	o__Cloacimonadales	f__W27	g__W27	0	0	0	0	0.037123824	0.027174516
d__Bacteria	p__Firmicutes	c__Incertae_Sedis	o__DTU014	f__DTU014	g__DTU014	0	0	0	0	0	0.000155283
d__Bacteria	p__Bacteroidota	c__Bacteroidia	o__Bacteroidales	f__Williamwhitmaniaceae	g__Acetobacteroides	0	0	0	0	0	0
d__Bacteria	p__Synergistota	c__Synergistia	o__Synergistales	f__Synergistaceae	g__Aminobacterium	0	0	0.003619723	0.011697689	0.000150638	0
d__Bacteria	p__Firmicutes	c__Clostridia	o__Peptostreptococcales-Tissierellales	f__Anaerovoracaceae	g__Anaerovorax	0.01590694	0.001670844	0.014610176	0.00445403	0.026512235	0.002240511
d__Bacteria	p__Bacteroidota	c__Bacteroidia	o__Bacteroidales	f__Rikenellaceae	g__dgA-11_gut_group	0	0	0	0	0	0
d__Bacteria	p__Firmicutes	c__Clostridia	o__Eubacteriales	f__Eubacteriaceae	g__Eubacterium	0	0	0	0	0	0
d__Archaea	p__Thermoplasmatota	c__Thermoplasmata	o__Methanomassiliicoccales	f__Methanomassiliicoccaceae	g__Methanomassiliicoccus	0	6.27E-05	0	0	0.020788003	0.006899
d__Bacteria	p__Firmicutes	c__Clostridia	o__Peptostreptococcales-Tissierellales	f__Sedimentibacteraceae	g__Sedimentibacter	0.000274784	0.000814536	0	7.03E-05	0.000117163	0
d__Bacteria	p__Bacteroidota	c__Bacteroidia	o__Bacteroidales	f__Bacteroidaceae	g__Bacteroides	0	0	0	0	0	0
d__Bacteria	p__Bacteroidota	c__Bacteroidia	o__Sphingobacteriales	f__Lentimicrobiaceae	g__Lentimicrobiaceae	0	0	0	0	0	0
d__Bacteria	p__Chloroflexi	c__Anaerolineae	o__Anaerolineales	f__Anaerolineaceae	g__Longilinea	0	0	0	0	0	0.000110916
d__Bacteria	p__Synergistota	c__Synergistia	o__Synergistales	f__Synergistaceae	g__Lactivibrio	0	0	0	0	0.003933318	0.002240511
d__Bacteria	p__Firmicutes	c__Clostridia	o__Christensenellales	f__Christensenellaceae	g__Christensenellaceae_R-7_group	0	0.001524645	0.006376714	0.006704487	0.007799685	0.000465849
d__Bacteria	p__Firmicutes	c__Clostridia	o__Clostridia	f__Gracilibacteraceae	g__Gracilibacter	0	0	0	0	0	0
d__Bacteria	p__Firmicutes	c__Clostridia	o__Peptostreptococcales-Tissierellales	f__Peptostreptococcales-Tissierellales	g__Soehngenia	0.003633255	0.01566416	0.005026351	0.002156688	0	0
d__Archaea	p__Halobacterota	c__Methanosarcinia	o__Methanosarciniales	f__Methanosaetaceae	g__Methanosaeta	0	0	0	0	0	0.000155283
d__Bacteria	p__Patescibacteria	c__Dojkabacteria	o__Dojkabacteria	f__Dojkabacteria	g__Dojkabacteria	0	0	0	0	0	0
d__Bacteria	p__Proteobacteria	c__Gammaproteobacteria	o__Pseudomonadales	f__Pseudomonadaceae	g__Pseudomonas	0.000366379	0.000396825	0	0.000398518	0	0
d__Bacteria	p__Bacteroidota	c__Bacteroidia	o__Bacteroidales	f__Dysgonomonadaceae	g__uncultured	0	0	0	0	0	0
d__Bacteria	p__Firmicutes	c__Bacilli	o__Erysipelotrichales	f__Erysipelatoclostridiaceae	g__UCG-004	0	0	0	0	0	0
d__Archaea	p__Thermoplasmatota	c__Thermoplasmata	o__Methanomassiliicoccales	f__Methanomethylophilaceae	g__Candidatus_Methanogranum	0	4.18E-05	0	0	0	0
d__Bacteria	p__Bacteroidota	c__Bacteroidia	o__Bacteroidales	f__Prolixibacteraceae	g__uncultured	0	0	0	0	0	0
d__Bacteria	p__Actinobacteriota	c__Coriobacteriia	o__OPB41	f__OPB41	g__OPB41	0.000122126	0	0.002888276	0.01399503	8.37E-05	0
d__Bacteria	p__Caldatribacteriota	c__Caldatribacteriia	o__Caldatribacteriales	f__Caldatribacteriaceae	g__Candidatus_Caldatribacterium	0	0	0.00018755	0	0.000251063	0.000244016
d__Bacteria	p__Firmicutes	c__Desulfitobacteriia	o__Desulfitobacteriales	f__uncultured	g__uncultured	0.003633255	0.012719298	0.000918997	0.005063529	0	0
d__Bacteria	p__Firmicutes	c__Clostridia	o__Peptococcales	f__Peptococcaceae	g__uncultured	0.000305316	0.001086048	0.001913014	0.011978996	0.000602551	0
d__Bacteria	p__Cloacimonadota	c__Cloacimonadia	o__Cloacimonadales	f__MSBL8	g__MSBL8	0	0	0.001669199	0.011721131	0	0
d__Bacteria	p__Firmicutes	c__Clostridia	o__Peptostreptococcales-Tissierellales	f__Anaerovoracaceae	g__uncultured	0	6.27E-05	0.011665635	0.002414553	0	0
d__Bacteria	p__Bacteroidota	c__Bacteroidia	o__Bacteroidales	f__uncultured	g__uncultured	0	0	0	0	0	0
d__Bacteria	p__Chloroflexi	c__Anaerolineae	o__SBR1031	f__SBR1031	g__SBR1031	0	0	0	0	0.00033475	0.000554582
d__Bacteria	p__Acidobacteriota	c__Subgroup_22	o__Subgroup_22	f__Subgroup_22	g__Subgroup_22	0	0	0	0	0	0
d__Bacteria	p__Spirochaetota	c__Spirochaetia	o__Spirochaetales	f__Spirochaetaceae	g__Sphaerochaeta	0	0	0.010052702	0.003797646	0.000836876	0.000221833
d__Bacteria	p__Bacteroidota	c__Bacteroidia	o__Bacteroidales	f__009E01-B-SD-P15	g__009E01-B-SD-P15	0	0	0	0	0.005037994	0.009827192

d__Bacteria	p__Desulfobacterota	c__Syntrophia	o__Syntrophales	f__Smithellaceae	g__Smithella	0	0	0	0	0	0
d__Bacteria	p__Nitrospirota	c__Thermodesulfovibrionia	o__uncultured	f__uncultured	g__uncultured	0	0	0	0	0.003464667	0.009649726
d__Bacteria	p__Firmicutes	c__Negativicutes	o__Veillonellales-Selenomonadales	f__Selenomonadaceae	g__Propionispira	0	0	0	0	0	0
d__Bacteria	p__Bacteroidota	c__Bacteroidia	o__Bacteroidales	f__Rikenellaceae	g__Bact-08	0	0	0	0	0	0
d__Bacteria	p__Chloroflexi	c__Anaerolineae	o__Anaerolineales	f__Anaerolineaceae	g__Leptolinea	0	0	0	0	0.000786664	0.000155283
d__Bacteria	p__Firmicutes	c__Clostridia	o__Oscillospirales	f__Ruminococcaceae	g__Anaerofilum	0	0	0	0	0	0
d__Bacteria	p__Armatimonadota	c__Chthonomonadetes	o__Chthonomonadales	f__Chthonomonadales	g__Chthonomonadales	0	0	0	0	0.008569611	0.005168704
d__Bacteria	p__Latescibacterota	c__Latescibacteria	o__Latescibacterales	f__Latescibacteraceae	g__Candidatus_Latescibacter	0	0	0	0	0	0
d__Bacteria	p__Acidobacteriota	c__Aminicenantia	o__Aminicenantales	f__Aminicenantales	g__Aminicenantales	0	0	0	0	0.000485388	0.001264447
d__Bacteria	p__Thermotogota	c__Thermotogae	o__Petrotogales	f__Petrotogaceae	g__AUTHM297	0	0	0	0	0.000368225	0.001685929
d__Bacteria	p__Desulfobacterota	c__Syntrophia	o__Syntrophales	f__Syntrophaceae	g__Syntrophus	0	0	0	0	0.0001339	0
d__Bacteria	p__Firmicutes	c__Thermoanaerobacteria	o__Thermoanaerobacterales	f__SRB2	g__SRB2	0.005404085	0.007790309	0	0	0	0
d__Bacteria	p__Thermotogota	c__Thermotogae	o__Kosmotogales	f__Kosmotogaceae	g__Mesotoga	0	0	0.005926593	0.007759389	0.000719713	0.000288383
d__Bacteria	p__Desulfobacterota	c__Syntrophia	o__Syntrophales	f__uncultured	g__uncultured	0	0	0	0	0.000150638	0.000643315
d__Bacteria	p__Firmicutes	c__Desulfitobacteriia	o__Desulfitobacteriales	f__Desulfitobacteriales	g__Candidatus_Dichloromethanomonas	0	0	0.001369118	0.007571851	0	0
d__Bacteria	p__Proteobacteria	c__Gammaproteobacteria	o__Aeromonadales	f__Aeromonadaceae	g__Aeromonas	0	0	0	0	0	0
d__Bacteria	p__Firmicutes	c__Negativicutes	o__Veillonellales-Selenomonadales	f__Sporomusaceae	g__uncultured	0	0	0	0	0.007247347	0.001663746
d__Bacteria	p__Caldisericota	c__Caldisericia	o__Caldisericales	f__WCHB1-02	g__WCHB1-02	0	0	0	0	0	0
d__Bacteria	p__Firmicutes	c__Syntrophomonadia	o__Syntrophomonadales	f__Syntrophomonadaceae	g__Syntrophomonas	0	0	0	0	0.001154889	8.87E-05
d__Bacteria	p__Firmicutes	c__Negativicutes	o__Acidaminococcales	f__Acidaminococcaceae	g__Acidaminococcus	0	0	0	0	0	0
d__Bacteria	p__Firmicutes	c__Clostridia	o__Oscillospirales	f__Oscillospiraceae	g__Oscillibacter	0	0	0	0	0	0
d__Bacteria	p__Synergistota	c__Synergistia	o__Synergistales	f__Synergistaceae	g__EBM-39	0	0	0.005457717	0.005930892	0.000602551	0.000221833
d__Archaea	p__Halobacterota	c__Methanomicrobia	o__Methanomicrobiales	f__Methanoregulaceae	g__Methanoregula	0	0	0	0	0	0
d__Bacteria	p__Verrucomicrobiota	c__Lentisphaeria	o__Victivallales	f__GWF2-44-16	g__GWF2-44-16	0	0	0	0	0	0
d__Bacteria	p__Firmicutes	c__Clostridia	o__Oscillospirales	f__Oscillospiraceae	g__NK4A214_group	0	0	0	0	0	0
d__Bacteria	p__Cloacimonadota	c__Cloacimonadia	o__Cloacimonadales	f__PBS-18	g__PBS-18	0	0	0	0	0	4.44E-05
d__Bacteria	p__Desulfobacterota	c__Desulfovibrionia	o__Desulfovibrionales	f__Desulfovibrionaceae	g__Desulfocurvus	0	0	0.001012772	0.005086971	0	0
d__Bacteria	p__Cloacimonadota	c__Cloacimonadia	o__Cloacimonadales	__	__	0	0	0	0	0	0
d__Bacteria	p__Chloroflexi	c__Anaerolineae	o__RBG-13-54-9	f__RBG-13-54-9	g__RBG-13-54-9	0	0	0	0	0	0
d__Bacteria	p__WPS-2	c__WPS-2	o__WPS-2	f__WPS-2	g__WPS-2	0	0.000543024	0	0	0	0
d__Bacteria	p__Bacteroidota	c__Bacteroidia	o__Bacteroidales	f__Muribaculaceae	g__Muribaculaceae	0	0	0	0	0	0
d__Bacteria	p__Firmicutes	c__Negativicutes	o__Veillonellales-Selenomonadales	f__Sporomusaceae	g__Pelosinus	0	0.000856307	0	0	0	0
d__Bacteria	p__Desulfobacterota	c__Syntrophorhabdia	o__Syntrophorhabdales	f__Syntrophorhabdaceae	g__Syntrophorhabdus	0	0	0	0	0.0002678	0.000931698
d__Bacteria	p__Proteobacteria	c__Gammaproteobacteria	o__Pseudomonadales	f__Moraxellaceae	g__Acinetobacter	0	0	0	0	0	0
d__Archaea	p__Halobacterota	c__Methanomicrobia	o__Methanomicrobiales	f__Methanoregulaceae	g__Methanolinea	0	0	0	0	0	0
d__Bacteria	p__Actinobacteriota	c__Actinobacteria	o__Corynebacteriales	f__Mycobacteriaceae	g__Mycobacterium	0	0	0	0	0	0
d__Bacteria	p__Hydrogenedentes	c__Hydrogenedentia	o__Hydrogenedentiales	f__Hydrogenedensaceae	g__YC-ZSS-LKJ63	0	0	0	0	0	0
d__Bacteria	p__Firmicutes	c__Negativicutes	o__Acidaminococcales	f__Acidaminococcaceae	g__uncultured	0	0	0	0	0	0
d__Bacteria	p__Actinobacteriota	c__WCHB1-81	o__WCHB1-81	f__WCHB1-81	g__WCHB1-81	0	0	9.38E-05	0.000140654	0.00020085	0.000354932
d__Bacteria	p__Chloroflexi	c__Anaerolineae	o__Anaerolineales	f__Anaerolineaceae	g__Flexilinea	0	0	0	0	6.70E-05	0
d__Archaea	p__Euryarchaeota	c__Methanobacteria	o__Methanobacteriales	f__Methanobacteriaceae	g__Methanosphaera	0	0	0	0	0	0
d__Bacteria	p__Patescibacteria	c__Microgenomatia	o__Candidatus_Woesebacteria	f__Candidatus_Woesebacteria	g__Candidatus_Woesebacteria	0	0	0	0	0	0
d__Bacteria	p__Bacteroidota	c__Kapabacteria	o__Kapabacteriales	f__Kapabacteriales	g__Kapabacteriales	0	0	0	0	0.000301275	0
d__Bacteria	p__Desulfobacterota	c__Syntrophobacteria	o__Syntrophobacterales	f__Syntrophobacteraceae	g__Syntrophobacter	0	0	0	0	0	0.000266199
d__Archaea	p__Euryarchaeota	c__Methanobacteria	o__Methanobacteriales	f__Methanobacteriaceae	g__Methanobrevibacter	0	0	0	0	0	0
d__Bacteria	p__Caldisericota	c__Caldisericia	o__Caldisericales	f__LF045	g__LF045	0	0	0	0	0	0
d__Bacteria	p__Firmicutes	c__Desulfotomaculia	o__Desulfotomaculales	f__Desulfotomaculales	g__Pelotomaculum	0	0	0	0	0	0
d__Bacteria	p__Actinobacteriota	c__Actinobacteria	o__Micrococcales	f__Microbacteriaceae	g__Galbitalea	0	0	0	0	0	0
d__Bacteria	p__Chloroflexi	c__Anaerolineae	o__Anaerolineales	f__Anaerolineaceae	g__Anaerolineaceae	0	0	0	0	0	0
d__Bacteria	p__Firmicutes	c__Clostridia	o__Eubacteriales	f__Anaerofustaceae	g__Anaerofustis	0	0	0	0	0.003079704	0.000421482
d__Bacteria	p__Bacteroidota	c__SJA-28	o__SJA-28	f__SJA-28	g__SJA-28	0	0	0	0	0.001924815	6.65E-05
d__Bacteria	p__SAR324_clade(Mar	c__SAR324_clade(Marine_gr	o__SAR324_clade(Marine_group_B)	f__SAR324_clade(Marine_group_B)	g__SAR324_clade(Marine_group_B)	0	0	0	0	0	0
d__Bacteria	p__Synergistota	c__Synergistia	o__Synergistales	f__Synergistaceae	__	0.002869966	0.001315789	0	0	0	0
d__Bacteria	p__Actinobacteriota	c__Thermoleophilia	o__Gaiellales	f__uncultured	g__uncultured	0	0	0	0	0	0
d__Bacteria	p__Firmicutes	c__Clostridia	o__Clostridia	f__Hungateiclostridiaceae	g__Ruminiclostridium	0	0.000125313	0	0	0	0
d__Bacteria	p__Chloroflexi	c__Anaerolineae	o__Anaerolineales	f__Anaerolineaceae	g__Pelolinea	0	0	0	0	0.002644529	0.001020431
d__Bacteria	p__Firmicutes	c__Clostridia	o__Clostridiales	f__Clostridiaceae	g__Proteiniclasticum	0.000366379	0.000355054	0	0	0.000351488	0
d__Bacteria	p__Firmicutes	c__Clostridia	o__Peptostreptococcales-Tissierellales	f__Peptostreptococcaceae	g__Romboutsia	0	0	0	0	0	0
d__Bacteria	p__Acidobacteriota	c__Acidobacteriae	__	__	__	0	0	0	0	0	0
d__Bacteria	p__Firmicutes	c__Syntrophomonadia	o__Syntrophomonadales	f__Syntrophomonadaceae	g__uncultured	0	0	0.000525141	0.000117211	0.00162354	0.000155283
d__Bacteria	p__Firmicutes	c__Clostridia	o__Oscillospirales	f__Oscillospiraceae	g__uncultured	0	0	0	0	0	0
d__Bacteria	p__Firmicutes	c__Clostridia	o__Christensenellales	f__Christensenellaceae	g__uncultured	0	0	0	0	0	0.000110916
d__Bacteria	p__Verrucomicrobiota	c__Verrucomicrobiae	o__Pedosphaerales	f__Pedosphaeraceae	g__ADurb.Bin063-1	0	0	0	0	0	0
d__Bacteria	p__Bacteroidota	c__Bacteroidia	o__Bacteroidales	f__Prolixibacteraceae	g__Meniscus	0	0	0	0	0	0
d__Bacteria	p__Zixibacteria	c__Zixibacteria	o__Zixibacteria	f__Zixibacteria	g__Zixibacteria	0	0	0	0	0	0
d__Bacteria	p__Firmicutes	c__Clostridia	o__Oscillospirales	f__Oscillospiraceae	g__Intestinimonas	0	0	0.00234438	0.001218998	0	0.0001331
d__Bacteria	p__Firmicutes	c__Clostridia	o__Clostridia_UCG-014	f__Clostridia_UCG-014	g__Clostridia_UCG-014	0	0	0	0	0	0
d__Bacteria	p__Proteobacteria	c__Alphaproteobacteria	o__Rhizobiales	f__Hyphomicrobiaceae	g__Hyphomicrobium	0	0	0	0	0	0
d__Bacteria	p__Firmicutes	c__Clostridia	o__Peptostreptococcales-Tissierellales	f__Peptostreptococcales-Tissierellales	g__uncultured	0	0	0.00223185	0.000609499	0	0
d__Bacteria	p__Firmicutes	c__Negativicutes	o__Veillonellales-Selenomonadales	f__Sporomusaceae	g__Anaeromusa-Anaeroarcus	0	0	0	0	0	0
d__Bacteria	p__Firmicutes	c__Clostridia	o__Lachnospirales	f__Lachnospiraceae	g__Anaerostignum	0	0.002192982	0	0.000843921	0.000117163	4.44E-05
d__Bacteria	p__Proteobacteria	c__Alphaproteobacteria	o__Rhizobiales	f__Rhizobiales_Incertae_Sedis	g__uncultured	0	0	0	0	0	0
d__Bacteria	p__Verrucomicrobiota	c__Verrucomicrobiae	o__Pedosphaerales	f__Pedosphaeraceae	g__Pedosphaeraceae	0	0	0	0	0	0
d__Bacteria	p__Spirochaetota	c__Brachyspirae	o__Brachyspirales	f__Brachyspirales_Incertae_Sedis	g__Exilispira	0	0	0	0	0	0
d__Bacteria	p__Bacteroidota	c__Bacteroidia	o__Bacteroidales	f__Rikenellaceae	g__Alistipes	0	0	0.000206305	0.002133246	0	0.0001331
d__Bacteria	p__Campilobacterota	c__Campylobacteria	o__Campylobacterales	f__Arcobacteraceae	g__Pseudarcobacter	0	0	0	0	0	0
d__Bacteria	p__Fermentibacterota	c__Fermentibacteria	o__Fermentibacterales	f__Fermentibacteraceae	g__Fermentibacteraceae	0	0	0	0	0	0
d__Bacteria	p__Thermotogota	c__Thermotogae	o__Petrotogales	f__Petrotogaceae	g__SC103	0	0	0	0	0	0
d__Bacteria	p__Bacteroidota	c__Bacteroidia	o__Bacteroidales	f__Dysgonomonadaceae	g__Fermentimonas	0	0	9.38E-05	0	0	0

d__Bacteria	p__Acidobacteriota	c__c5LKS83	o__c5LKS83	f__c5LKS83	g__c5LKS83	0	0	0	0	0	0
d__Bacteria	p__Chloroflexi	c__Anaerolineae	o__ADurb.Bin180	f__ADurb.Bin180	g__ADurb.Bin180	0	0	0	0	0	0
d__Bacteria	p__Firmicutes	c__Clostridia	o__Oscillospirales	f__Ruminococcaceae	g__Candidatus_Soleaferrea	0	0	0	0	0	0
d__Archaea	p__Halobacterota	c__Methanomicrobia	o__Methanomicrobiales	f__Methanomicrobiaceae	g__Methanoculleus	0	0	0.001650444	0.000351634	0	0
d__Bacteria	p__Elusimicrobiota	c__Endomicrobia	o__Endomicrobiales	f__Endomicrobiaceae	g__Endomicrobium	0	0.00037594	0	0	0.001238577	0.000510215
d__Bacteria	p__Chloroflexi	c__Anaerolineae	o__Anaerolineales	f__Anaerolineaceae	g__Bellilinea	0	0	0	0	0	0
d__Bacteria	p__Firmicutes	c__D8A-2	o__D8A-2	f__D8A-2	g__D8A-2	0	0	0	0	0	0
d__Bacteria	p__Firmicutes	c__Clostridia	o__Clostridia	f__Hungateiclostridiaceae	g__HN-HF0106	0	0	0	0.000117211	0	0
d__Bacteria	p__Synergistota	c__Synergistia	o__Synergistales	f__Synergistaceae	g__Aminomonas	0	0	0	0	0	0
d__Bacteria	p__Planctomycetota	c__Planctomycetes	o__Pirellulales	f__Pirellulaceae	g__uncultured	0	0	0	0	0	0
d__Bacteria	p__Proteobacteria	c__Alphaproteobacteria	o__Rhodobacterales	f__Rhodobacteraceae	g__Rhodobacter	0	0	0	0	0	0
d__Bacteria	p__Firmicutes	c__Clostridia	o__Clostridiales	f__Clostridiaceae	g__Clostridium_sensu_stricto_1	0	0	0	0	0	0
d__Bacteria	p__Calditrichota	c__Calditrichia	o__Calditrichales	f__Calditrichaceae	g__Calditrichaceae	0	0	0	0	0	0
d__Bacteria	p__Bacteroidota	c__Ignavibacteria	o__Ignavibacteriales	f__BSV40	g__BSV40	0	0	0	0	0	0
d__Bacteria	p__Patescibacteria	c__Berkelbacteria	o__Berkelbacteria	f__Berkelbacteria	g__Berkelbacteria	0	0	0	0	0	0
d__Bacteria	p__Firmicutes	__	__	__	__	0.00137392	0	0	0	0	0
d__Bacteria	p__Actinobacteriota	c__Actinobacteria	o__Micrococcales	f__Intrasporangiaceae	g__Ornithinibacter	0	0	0	0	0	0
d__Bacteria	p__Proteobacteria	c__Gammaproteobacteria	o__Burkholderiales	f__Comamonadaceae	g__Acidovorax	0	0	0	0	0	0
d__Bacteria	p__Spirochaetota	c__V2072-189E03	o__V2072-189E03	f__V2072-189E03	g__V2072-189E03	0	0	0	0	0	0
d__Bacteria	p__Hydrogenedentes	c__Hydrogenedentia	o__Hydrogenedentiales	f__Hydrogenedensaceae	g__Hydrogenedensaceae	0	0	0.000206305	0	0	0
d__Bacteria	p__Chloroflexi	c__Anaerolineae	o__uncultured	f__uncultured	g__uncultured	0	0	0	0	0	0
d__Bacteria	__	__	__	__	__	0.000213721	8.35E-05	0	0	0.001322264	0.000754231
d__Bacteria	p__Firmicutes	c__Clostridia	o__Clostridia_vadinBB60_group	f__Clostridia_vadinBB60_group	g__Clostridia_vadinBB60_group	0	0	0	0	0	0
d__Bacteria	p__Patescibacteria	__	__	__	__	0	0	0	0	0	0
d__Bacteria	p__Proteobacteria	c__Alphaproteobacteria	o__Sphingomonadales	f__Sphingomonadaceae	g__uncultured	0	0	0	0	0	0
d__Bacteria	p__Thermotogota	c__Thermotogae	o__Thermotogales	f__Fervidobacteriaceae	g__Fervidobacterium	0	0	0	0	0	0.000399299
d__Bacteria	p__uncultured	c__uncultured	o__uncultured	f__uncultured	g__uncultured	0	0	0	0	0	0
d__Bacteria	p__Bacteroidota	c__Bacteroidia	o__Flavobacteriales	f__Flavobacteriaceae	g__Flavobacterium	0	0	0	0	0	0
d__Bacteria	p__Spirochaetota	c__Leptospirae	o__Leptospirales	f__Leptospiraceae	g__RBG-16-49-21	0	0	0	0	0	0
d__Bacteria	p__Proteobacteria	c__Gammaproteobacteria	o__Burkholderiales	f__Comamonadaceae	__	0	0	0	0	0	0
d__Bacteria	p__Latescibacterota	c__Latescibacterota	o__Latescibacterota	f__Latescibacterota	g__Latescibacterota	0	0	0	0	0	0
d__Bacteria	p__Patescibacteria	c__WWE3	o__WWE3	f__WWE3	g__WWE3	0	0	0	0	0	0
d__Bacteria	p__Proteobacteria	c__Alphaproteobacteria	o__Rhizobiales	f__Xanthobacteraceae	__	0	0	0	0	0	0
d__Bacteria	p__Firmicutes	c__Clostridia	o__Peptostreptococcales-Tissierellales	f__Anaerovoracaceae	g__[Eubacterium]_brachy_group	0	0	0	0	0.000251063	0.000110916
d__Bacteria	p__Proteobacteria	c__Alphaproteobacteria	o__Rhodobacterales	f__Rhodobacteraceae	g__Thioclava	0	0	0	0	0	0
d__Bacteria	p__Bacteroidota	c__Bacteroidia	o__Bacteroidales	f__Tannerellaceae	g__Macellibacteroides	0	8.35E-05	0	0	0	0
d__Bacteria	p__Firmicutes	c__Clostridia	o__Clostridia	f__Hungateiclostridiaceae	g__Ercella	0	0	0.00018755	0	0	0
d__Bacteria	p__Acidobacteriota	c__Acidobacteriae	o__TSBb06	f__TSBb06	g__TSBb06	0	0	0	0	0.001121414	0
d__Bacteria	p__Bacteroidota	c__Bacteroidia	o__Bacteroidales	f__Prevotellaceae	g__Prevotellaceae_UCG-004	0	0	0	0	0	0
d__Bacteria	p__Planctomycetota	c__Phycisphaerae	o__Phycisphaerales	f__AKAU3564_sediment_group	g__AKAU3564_sediment_group	0	0	0	0	0	0
d__Bacteria	p__Firmicutes	c__Clostridia	o__Oscillospirales	f__Oscillospiraceae	__	0	0	0	0	0	0
d__Bacteria	p__Proteobacteria	c__Gammaproteobacteria	o__Burkholderiales	f__Burkholderiaceae	g__Lautropia	0	0	0	0	0	0
d__Bacteria	p__Firmicutes	c__Bacilli	o__Acholeplasmatales	f__Acholeplasmataceae	g__Acholeplasma	0	0.000271512	0	0	0	0
d__Bacteria	p__Desulfobacterota	c__Syntrophobacteria	o__Syntrophobacterales	f__Syntrophobacteraceae	__	0	0	0	0	0.001054464	0.000421482
d__Bacteria	p__Cloacimonadota	c__Cloacimonadia	o__Cloacimonadales	f__SHA-4	g__SHA-4	0	0	0	0	0	0
d__Bacteria	p__Bacteroidota	c__Bacteroidia	o__Bacteroidales	f__Rikenellaceae	__	0	0.001002506	0	0	0	0
d__Bacteria	p__Chloroflexi	c__Anaerolineae	o__Anaerolineales	f__Anaerolineaceae	g__Anaerolinea	0	0	0	0	0	0
d__Bacteria	p__Proteobacteria	c__Alphaproteobacteria	o__Rhodobacterales	f__Rhodobacteraceae	g__Defluviimonas	0	0	0	0	0	0
d__Bacteria	p__Chloroflexi	c__Chloroflexia	o__Thermomicrobiales	f__JG30-KF-CM45	g__JG30-KF-CM45	0	0	0	0	0	0
d__Bacteria	p__Proteobacteria	c__Gammaproteobacteria	o__Burkholderiales	f__Comamonadaceae	g__Rhodoferax	0	0	0	0	0	0
d__Bacteria	p__Proteobacteria	c__Gammaproteobacteria	o__Burkholderiales	f__Rhodocyclaceae	g__Dechlorobacter	0	0.000918964	0	0	0	0
d__Bacteria	p__Chloroflexi	c__Anaerolineae	o__SJA-15	f__SJA-15	g__SJA-15	0	0	0	0	0	0
d__Bacteria	p__Proteobacteria	c__Alphaproteobacteria	o__Rhizobiales	f__Xanthobacteraceae	g__Bradyrhizobium	0	0	0	0	0	0
d__Bacteria	p__Chloroflexi	c__Anaerolineae	o__Anaerolineales	f__Anaerolineaceae	g__Levilinea	0	0	0	0	0.000736451	0
d__Bacteria	p__Planctomycetota	c__Planctomycetes	o__Pirellulales	f__Pirellulaceae	g__Pir4_lineage	0	0	0	0	0	0
d__Bacteria	p__Proteobacteria	c__Alphaproteobacteria	o__Rhizobiales	f__Beijerinckiaceae	g__uncultured	0	0	0	0	0	0
d__Bacteria	p__Patescibacteria	c__Microgenomatia	o__Candidatus_Pacebacteria	f__Candidatus_Pacebacteria	g__Candidatus_Pacebacteria	0	0	0	0	0	0
d__Bacteria	p__Actinobacteriota	c__Actinobacteria	o__Corynebacteriales	f__Nocardiaceae	g__Rhodococcus	0	0	0	0	0	0
d__Bacteria	p__Actinobacteriota	c__Actinobacteria	o__Micrococcales	f__Microbacteriaceae	g__Microbacterium	0	0	0	0	0	0
d__Bacteria	p__Firmicutes	c__Bacilli	o__Erysipelotrichales	f__Erysipelotrichaceae	g__Turicibacter	0	0	0	0	0	0
d__Bacteria	p__Firmicutes	c__Clostridia	o__Oscillospirales	f__[Eubacterium]_coprostanoligenes_g	g__[Eubacterium]_coprostanoligenes_group	0	0	0	0	0	0
d__Archaea	p__Thermoplasmatota	c__Thermoplasmata	o__Methanomassiliicoccales	f__Methanomassiliicoccaceae	g__uncultured	0	0	0	0	0.000167375	0
d__Bacteria	p__Firmicutes	c__Clostridia	o__Oscillospirales	f__Oscillospirales	g__Hydrogenoanaerobacterium	0	0.000730994	0	0.000164096	0	0
d__Bacteria	p__Cyanobacteria	c__Vampirivibrionia	o__Obscuribacterales	f__Obscuribacteraceae	g__Candidatus_Obscuribacter	0	0	0	0	0	0
d__Bacteria	p__Sumerlaeota	c__Sumerlaeia	o__uncultured	f__uncultured	g__uncultured	0	0	0	0	0	0
d__Bacteria	p__Cloacimonadota	c__Cloacimonadia	o__Cloacimonadales	f__Cloacimonadaceae	g__Cloacimonadaceae	0	0.000710109	0	0	0	0
d__Bacteria	p__Actinobacteriota	c__Thermoleophilia	o__Solirubrobacterales	f__67-14	g__67-14	0	0	0	0	0	0
d__Bacteria	p__Synergistota	c__Synergistia	o__Synergistales	f__Synergistaceae	g__Aminivibrio	0	0	0	0	0	0
d__Bacteria	p__Firmicutes	c__Clostridia	o__Clostridia	f__Hungateiclostridiaceae	g__Fastidiosipila	0	0	0.000675181	0.000586057	0	0
d__Bacteria	p__Firmicutes	c__Clostridia	o__Oscillospirales	f__Ruminococcaceae	g__uncultured	0	0	0	0	0	0
d__Bacteria	p__Verrucomicrobiota	c__Omnitrophia	o__Omnitrophales	f__Omnitrophaceae	g__Candidatus_Omnitrophus	0	0	0	0	0	0
d__Bacteria	p__Bacteroidota	c__Bacteroidia	o__Bacteroidales	f__Paludibacteraceae	g__Paludibacter	0	0.000647452	0	0	0	0
d__Bacteria	p__Firmicutes	c__Clostridia	o__Clostridia	f__Gracilibacteraceae	__	0	0	0	0	0	0
d__Bacteria	p__Spirochaetota	c__Spirochaetia	o__Spirochaetales	f__Spirochaetaceae	g__Treponema	0	0	0	0	0	0
d__Bacteria	p__Actinobacteriota	c__Acidimicrobiia	o__Microtrichales	f__Ilumatobacteraceae	g__CL500-29_marine_group	0	0	0	0	0	0
d__Bacteria	p__Planctomycetota	c__Pla3_lineage	o__Pla3_lineage	f__Pla3_lineage	g__Pla3_lineage	0	0	0	0	0	0
d__Bacteria	p__Desulfobacterota	c__Desulfobulbia	o__Desulfobulbales	f__Desulfobulbaceae	g__Desulfobulbus	0	0.000605681	0	0	0	0
d__Bacteria	p__Spirochaetota	c__Spirochaetia	o__Spirochaetales	f__Spirochaetaceae	g__Spirochaeta	0	0	0	0	0.00033475	0
d__Bacteria	p__Proteobacteria	c__Alphaproteobacteria	o__Rhizobiales	f__Devosiaceae	g__Devosia	0	0	0	0	0	0

d__Bacteria	p__Acidobacteriota	c__Acidobacteriae	o__Acidobacteriae	f__Acidobacteriae	g__Paludibaculum	0	0	0	0	0	0
d__Bacteria	p__Proteobacteria	c__Alphaproteobacteria	o__Sphingomonadales	f__Sphingomonadaceae	g__Novosphingobium	0	0	0	0	0	0
d__Archaea	p__Halobacterota	c__Methanomicrobia	o__Methanomicrobiales	f__Methanomicrobiaceae	g__Methanofollis	0	0	0.00011253	0	0	0
d__Bacteria	p__Nitrospirota	c__4-29-1	o__4-29-1	f__4-29-1	g__4-29-1	0	0	0	0	0.000552338	8.87E-05
d__Bacteria	p__Proteobacteria	c__Alphaproteobacteria	o__Caulobacterales	f__Hyphomonadaceae	g__Hirschia	0	0	0	0	0	0
d__Bacteria	p__Acidobacteriota	c__Vicinamibacteria	o__Vicinamibacterales	f__uncultured	g__uncultured	0	0	0	0	0	0
d__Bacteria	p__Bacteroidota	c__Bacteroidia	o__Bacteroidales	f__Dysgonomonadaceae	g__Dysgonomonadaceae	0	0	0	0	0	0
d__Bacteria	p__Verrucomicrobiota	c__Chlamydiae	o__LD1-PA32	f__LD1-PA32	g__LD1-PA32	0	0	0	0	0	0
d__Bacteria	p__Firmicutes	c__Clostridia	o__Peptostreptococcales-Tissierellales	f__Peptostreptococcaceae	g__Acetoanaerobium	0	0	0	0	0	0
d__Bacteria	p__Firmicutes	c__Clostridia	o__Oscillospirales	f__Oscillospiraceae	g__UCG-002	0	0	0	0	0	0
d__Bacteria	p__Planctomycetota	c__Phycisphaerae	o__Pla1_lineage	f__Pla1_lineage	g__Pla1_lineage	0	0	0	0	0	0
d__Bacteria	p__Proteobacteria	c__Alphaproteobacteria	o__Caulobacterales	f__Hyphomonadaceae	g__SWB02	0	0	0	0	0	0
d__Bacteria	p__Proteobacteria	c__Alphaproteobacteria	o__Rhodobacterales	f__Rhodobacteraceae	g__Amaricoccus	0	0	0	0	0	0
d__Bacteria	p__Proteobacteria	c__Alphaproteobacteria	o__Rhizobiales	f__Hyphomicrobiaceae	g__Pedomicrobium	0	0	0	0	0	0
d__Bacteria	p__Firmicutes	c__Clostridia	o__Peptostreptococcales-Tissierellales	f__Peptostreptococcaceae	g__Intestinibacter	0	0	0	0	0	0
d__Bacteria	p__Verrucomicrobiota	c__Kiritimatiellae	o__WCHB1-41	f__WCHB1-41	g__WCHB1-41	0	0	0	0	0	0
d__Bacteria	p__Bacteroidota	c__Bacteroidia	o__Chitinophagales	f__Saprospiraceae	g__uncultured	0	0	0	0	0	0
d__Bacteria	p__Planctomycetota	c__Phycisphaerae	o__mle1-8	f__mle1-8	g__mle1-8	0	0	0	0	0	0
d__Bacteria	p__Planctomycetota	c__Planctomycetes	o__Planctomycetales	f__uncultured	g__uncultured	0	0	0	0	0	0
d__Bacteria	p__Proteobacteria	c__Alphaproteobacteria	o__Rhizobiales	f__Rhizobiaceae	g__Allorhizobium-Neorhizobium-Pararhizobium-Rhizobium	0.000183189	0	0	0	0	0
d__Bacteria	p__Caldatribacteriota	c__JS1	o__JS1	f__JS1	g__JS1	0	0	7.50E-05	0.000421961	0	0
d__Bacteria	p__Verrucomicrobiota	c__Verrucomicrobiae	o__Pedosphaerales	f__Pedosphaeraceae	g__DEV114	0	0	0	0	0	0
d__Bacteria	p__Firmicutes	c__Clostridia	o__Oscillospirales	__	__	0	0.000417711	0	0	0	0
d__Bacteria	p__Actinobacteriota	c__Actinobacteria	o__uncultured	f__uncultured	g__uncultured	0	0	0	0	0	0
d__Bacteria	p__Proteobacteria	c__Gammaproteobacteria	o__Burkholderiales	f__Rhodocyclaceae	g__Thauera	0	0	0	0	0	0
d__Bacteria	p__Verrucomicrobiota	c__Chlamydiae	o__Chlamydiales	f__Parachlamydiaceae	g__Neochlamydia	0	0	0	0	0	0
d__Bacteria	p__Proteobacteria	c__Gammaproteobacteria	o__Burkholderiales	f__Sutterellaceae	g__AAP99	0	0	0	0	0	0
d__Bacteria	p__Proteobacteria	c__Gammaproteobacteria	o__Enterobacterales	f__Enterobacteriaceae	g__Escherichia-Shigella	0	0	0	0	0	0
d__Archaea	p__Halobacterota	c__Methanomicrobia	o__Methanomicrobiales	f__Methanospirillaceae	g__Methanospirillum	0	0	0	0	0	0
d__Bacteria	p__Verrucomicrobiota	c__Lentisphaeria	o__Victivallales	f__Victivallaceae	g__Victivallaceae	0	0	0	0	0	0
d__Bacteria	p__Proteobacteria	c__Alphaproteobacteria	o__Rhizobiales	f__Pleomorphomonadaceae	g__Pleomorphomonas	0	0	0	0	0	0
d__Bacteria	p__Sumerlaeota	c__Sumerlaeia	o__Sumerlaeales	f__Sumerlaeaceae	g__Sumerlaea	0	0	0	0	0	0
d__Bacteria	p__Bacteroidota	c__Bacteroidia	o__Bacteroidales	f__Marinilabiliaceae	g__[Cytophaga]_xylanolytica_group	0	0.000396825	0	0	0	0
d__Bacteria	p__Bacteroidota	c__Bacteroidia	o__Bacteroidales	f__SB-5	g__SB-5	0	0	0	0	0	0
d__Bacteria	p__Planctomycetota	c__Planctomycetes	o__Planctomycetales	f__Rubinisphaeraceae	g__uncultured	0	0	0	0	0	0
d__Bacteria	p__Firmicutes	c__Clostridia	o__Lachnospirales	f__Lachnospiraceae	g__Lachnospiraceae_XPB1014_group	0	0	0	0	0	0
d__Bacteria	p__Firmicutes	c__Negativicutes	o__Veillonellales-Selenomonadales	f__Sporomusaceae	g__Sporomusa	0	0	0	0	0	0
d__Bacteria	p__Patescibacteria	c__Parcubacteria	o__Candidatus_Colwellbacteria	f__Candidatus_Colwellbacteria	g__Candidatus_Colwellbacteria	0	0	0	0	0	0
d__Bacteria	p__Caldisericota	c__Caldisericia	o__Caldisericales	f__TTA-B1	g__TTA-B1	0	0	0	0	0	0
d__Bacteria	p__Proteobacteria	c__Alphaproteobacteria	o__Rhizobiales	f__Xanthobacteraceae	g__uncultured	0	0	0	0	0	0
d__Bacteria	p__Proteobacteria	c__Alphaproteobacteria	o__Rhodobacterales	f__Rhodobacteraceae	__	0	0	0	0	0	0
d__Bacteria	p__Bacteroidota	c__Bacteroidia	o__Bacteroidales	f__Barnesiellaceae	g__uncultured	0	0	0	0	0	0
d__Bacteria	p__Firmicutes	c__Bacilli	o__Izemoplasmatales	f__Izemoplasmataceae	g__Izemoplasmataceae	0	0	0	0	0	0
d__Bacteria	p__Proteobacteria	c__Alphaproteobacteria	o__Rhizobiales	f__Devosiaceae	__	0	0	0	0	0	0
d__Bacteria	p__Proteobacteria	c__Gammaproteobacteria	o__Burkholderiales	f__Rhodocyclaceae	g__Dechloromonas	0	0	0	0	0	0
d__Bacteria	p__Proteobacteria	c__Gammaproteobacteria	o__Legionellales	f__Legionellaceae	g__Legionella	0	0	0	0	0	0
d__Bacteria	p__Armatimonadota	c__Fimbriimonadia	o__Fimbriimonadales	f__Fimbriimonadaceae	g__Fimbriimonadaceae	0	0	0	0	0	0
d__Bacteria	p__Bacteroidota	c__Bacteroidia	o__Bacteroidales	f__Rikenellaceae	g__Rikenellaceae_RC9_gut_group	0	0	0	0	0	0
d__Bacteria	p__Firmicutes	c__Negativicutes	o__Veillonellales-Selenomonadales	f__Veillonellaceae	g__Megasphaera	0	0	0	0	0	0
d__Bacteria	p__Firmicutes	c__Bacilli	o__Lactobacillales	f__Carnobacteriaceae	g__Trichococcus	0	0	0	0	0	0
d__Bacteria	p__Firmicutes	c__Clostridia	o__Oscillospirales	f__UCG-010	g__UCG-010	0	0	0	0	8.37E-05	0.000310566
d__Bacteria	p__Patescibacteria	c__Saccharimonadia	o__Saccharimonadales	f__Saccharimonadales	g__Saccharimonadales	0	0	0	0	0	0
d__Bacteria	p__Proteobacteria	c__Gammaproteobacteria	o__Burkholderiales	f__Comamonadaceae	g__Rhizobacter	0	0	0	0	0	0
d__Bacteria	p__Chloroflexi	c__Anaerolineae	o__Caldilineales	f__Caldilineaceae	g__uncultured	0	0	0	0	0	0
d__Bacteria	p__Desulfobacterota	c__uncultured	o__uncultured	f__uncultured	g__uncultured	0	0	0	0	0	0
d__Bacteria	p__Proteobacteria	c__Alphaproteobacteria	o__uncultured	f__uncultured	g__uncultured	0	0	0	0	0	0
d__Bacteria	p__Firmicutes	c__Clostridia	o__Oscillospirales	f__Ruminococcaceae	g__Caproiciproducens	0	0	0	0	0	0
d__Bacteria	p__Chloroflexi	c__Dehalococcoidia	o__FS117-23B-02	f__FS117-23B-02	g__FS117-23B-02	0	0	0	0	0	0
d__Bacteria	p__Firmicutes	c__Clostridia	o__Monoglobales	f__Monoglobaceae	g__Monoglobus	0	4.18E-05	0	0	0	0
d__Bacteria	p__Planctomycetota	c__Planctomycetes	o__Planctomycetales	f__Rubinisphaeraceae	g__SH-PL14	0	0	0	0	0	0
d__Bacteria	p__Bdellovibrionota	c__Oligoflexia	o__0319-6G20	f__0319-6G20	g__0319-6G20	0	0	0	0	0	0
d__Bacteria	p__Verrucomicrobiota	c__Chlamydiae	o__Chlamydiales	f__Parachlamydiaceae	g__Candidatus_Protochlamydia	0	0	0	0	0	0
d__Bacteria	p__Proteobacteria	c__Alphaproteobacteria	o__Micropepsales	f__Micropepsaceae	g__uncultured	0	0	0	0	0	0
d__Bacteria	p__WS4	c__WS4	o__WS4	f__WS4	g__WS4	0	0	0	0	0	0
d__Bacteria	p__Actinobacteriota	c__Actinobacteria	o__PeM15	f__PeM15	g__PeM15	0	0	0	0	0	0
d__Bacteria	p__Bacteroidota	c__Bacteroidia	o__Chitinophagales	f__Chitinophagaceae	g__Ferruginibacter	0	0	0	0	0	0
d__Bacteria	p__Firmicutes	c__Clostridia	o__Oscillospirales	f__Oscillospiraceae	g__Sporobacter	0	0	0	0	0.0002678	0
d__Bacteria	p__Chloroflexi	c__Anaerolineae	o__MSB-5E12	f__MSB-5E12	g__MSB-5E12	0	0	0	0	0.00020085	0.000266199
d__Bacteria	p__Acidobacteriota	c__Holophagae	o__Holophagales	f__Holophagaceae	g__Holophaga	0	0	0	0	0	0
d__Bacteria	p__Campilobacterota	c__Campylobacteria	o__Campylobacterales	f__Arcobacteraceae	g__Arcobacter	0	0	0	0	0	0
d__Bacteria	p__Synergistota	c__Synergistia	o__Synergistales	f__Synergistaceae	g__Cloacibacillus	0	0	0	0	0	0
d__Bacteria	p__Actinobacteriota	c__Acidimicrobiia	o__IMCC26256	f__IMCC26256	g__IMCC26256	0	0	0	0	0	0
d__Bacteria	p__Marinimicrobia_(S	c__Marinimicrobia_(SAR406	o__Marinimicrobia_(SAR406_clade)	f__Marinimicrobia_(SAR406_clade)	g__Marinimicrobia_(SAR406_clade)	0	0	0	0	0	0
d__Bacteria	p__Proteobacteria	c__Alphaproteobacteria	o__Rhizobiales	f__Hyphomicrobiaceae	__	0	0	0	0	0	0
d__Bacteria	p__Chloroflexi	c__Anaerolineae	o__Anaerolineales	f__Anaerolineaceae	g__ADurb.Bin120	0	0	0.00018755	0.000257865	0	0
d__Bacteria	p__Proteobacteria	c__Alphaproteobacteria	o__Rhizobiales	f__Rhizobiaceae	g__Mesorhizobium	0	0.00018797	0	0	0	0
d__Bacteria	p__Proteobacteria	c__Alphaproteobacteria	o__Rhodobacterales	f__Rhodobacteraceae	g__Paracoccus	0	0	0	0	0	0
d__Bacteria	p__Fibrobacterota	c__Fibrobacteria	o__Fibrobacterales	f__Fibrobacteraceae	g__Fibrobacter	0	0	0	0	0	0
d__Bacteria	p__Proteobacteria	c__Alphaproteobacteria	o__Rhizobiales	f__Rhizobiaceae	__	0	0	0	0	0	0

d__Bacteria	p__Proteobacteria	c__Alphaproteobacteria	o__Rhizobiales	f__Xanthobacteraceae	g__Pseudorhodoplanes	0	0	0	0	0	0
d__Bacteria	p__Firmicutes	c__Clostridia	o__Christensenellales	f__Christensenellaceae	__	0	0	0	0	0	0
d__Bacteria	p__Caldisericota	c__Caldisericia	o__Caldisericales	f__Caldisericaceae	g__Caldisericum	0	0	0	0	0	0
d__Bacteria	p__Firmicutes	c__Syntrophomonadia	o__Syntrophomonadales	f__Syntrophomonadaceae	g__Pelospora	0	0	0	0	0	0
d__Bacteria	p__Patescibacteria	c__Parcubacteria	o__Candidatus_Nomurabacteria	f__Candidatus_Nomurabacteria	g__Candidatus_Nomurabacteria	0	0	0	0	0	0
d__Bacteria	p__Planctomycetota	c__Pla4_lineage	o__Pla4_lineage	f__Pla4_lineage	g__Pla4_lineage	0	0	0	0	0	0
d__Bacteria	p__Actinobacteriota	c__Actinobacteria	o__Corynebacteriales	f__Nocardiaceae	g__Gordonia	0	0	0	0	0	0
d__Archaea	p__Euryarchaeota	c__Thermococci	o__Methanofastidiosales	f__Methanofastidiosaceae	g__Candidatus_Methanofastidiosum	0	0	0	0	0	0
d__Bacteria	p__Chloroflexi	c__Anaerolineae	o__Anaerolineales	f__Anaerolineaceae	g__Anaerolineaceae_UCG-001	0	0	0	0	0	0
d__Bacteria	p__Verrucomicrobiota	c__Verrucomicrobiae	o__LD1-PB3	f__LD1-PB3	g__LD1-PB3	0	0	0	0	0	0
d__Bacteria	p__Proteobacteria	c__Alphaproteobacteria	o__Sphingomonadales	f__Sphingomonadaceae	g__Sphingopyxis	0	0	0	0	0	0
d__Bacteria	p__Bacteroidota	c__Ignavibacteria	o__Ignavibacteriales	f__SR-FBR-L83	g__SR-FBR-L83	0	0	0	0	0	0
d__Bacteria	p__Firmicutes	c__Clostridia	o__Oscillospirales	f__uncultured	g__uncultured	0	0	0	0	0	0
d__Bacteria	p__Proteobacteria	c__Gammaproteobacteria	o__Burkholderiales	f__Rhodocyclaceae	g__Azospira	0.000213721	0.00018797	0	0	0	0
d__Bacteria	p__Proteobacteria	c__Alphaproteobacteria	o__Rhizobiales	f__Hyphomicrobiaceae	g__uncultured	0	0	0	0	0	0
d__Bacteria	p__Proteobacteria	c__Gammaproteobacteria	o__Burkholderiales	f__SC-I-84	g__SC-I-84	0	0	0	0	0	0
d__Bacteria	p__Actinobacteriota	c__Actinobacteria	o__Propionibacteriales	f__Propionibacteriaceae	g__Brooklawnia	0	0	0	0	0	0
d__Bacteria	p__Campilobacterota	c__Campylobacteria	o__Campylobacterales	f__Sulfurimonadaceae	g__Sulfuricurvum	0	0	0	0	0	0
d__Bacteria	p__Firmicutes	c__Clostridia	o__Clostridiales	f__Clostridiaceae	g__Youngiibacter	0	0	0	0	0	0
d__Bacteria	p__Patescibacteria	c__ABY1	o__Candidatus_Kerfeldbacteria	f__Candidatus_Kerfeldbacteria	g__Candidatus_Kerfeldbacteria	0	0	0	0	0	0
d__Bacteria	p__Planctomycetota	c__Planctomycetes	o__Pirellulales	f__Pirellulaceae	g__Pirellula	0	0	0	0	0	0
d__Bacteria	p__Bacteroidota	c__Bacteroidia	o__Bacteroidales	__	__	0	0	0	0	0	0
d__Bacteria	p__Patescibacteria	c__Microgenomatia	o__Candidatus_Collierbacteria	f__Candidatus_Collierbacteria	g__Candidatus_Collierbacteria	0	0	0	0	0	0
d__Bacteria	p__Planctomycetota	c__Phycisphaerae	o__CCM11a	f__CCM11a	g__CCM11a	0	0	0	0	0	0
d__Bacteria	p__Planctomycetota	c__Planctomycetes	o__Isosphaerales	f__Isosphaeraceae	g__uncultured	0	0	0	0	0	0
d__Bacteria	p__Bacteroidota	c__Ignavibacteria	o__Ignavibacteriales	f__Melioribacteraceae	g__IheB3-7	0	0	0	0	0	0
d__Bacteria	p__Firmicutes	c__Clostridia	o__Clostridia	f__Hungateiclostridiaceae	g__Saccharofermentans	0	0	0	0	0	0
d__Bacteria	p__Proteobacteria	c__Alphaproteobacteria	o__Dongiales	f__Dongiaceae	g__Dongia	0	0	0	0	0	0
d__Bacteria	p__Proteobacteria	c__Gammaproteobacteria	o__Burkholderiales	f__Rhodocyclaceae	g__Ferribacterium	0	0	0	0	0	0
d__Bacteria	p__Acidobacteriota	c__Holophagae	o__Holophagales	f__Holophagaceae	__	0	0	0	0	0	0
d__Bacteria	p__Actinobacteriota	c__Acidimicrobiia	o__Microtrichales	f__uncultured	g__uncultured	0	0	0	0	0	0
d__Bacteria	p__Desulfobacterota	c__Desulfuromonadia	o__Geobacterales	f__Geobacteraceae	g__Geobacter	0	0	0	0	0	0
d__Bacteria	p__Proteobacteria	c__Alphaproteobacteria	o__Rhizobiales	f__Beijerinckiaceae	g__Bosea	0	0	0	0	0	0
d__Bacteria	p__Proteobacteria	c__Alphaproteobacteria	o__Rhizobiales	f__Methyloligellaceae	g__uncultured	0	0	0	0	0	0
d__Bacteria	p__Proteobacteria	c__Alphaproteobacteria	o__Sphingomonadales	f__Sphingomonadaceae	g__Sphingosinicella	0	0	0	0	0	0
d__Bacteria	p__Proteobacteria	c__Gammaproteobacteria	o__Cellvibrionales	f__Spongiibacteraceae	g__BD1-7_clade	0	0	0	0	0	0
d__Bacteria	p__Firmicutes	c__Clostridia	__	__	__	0	0	0	0	0	0
d__Bacteria	p__Actinobacteriota	c__Actinobacteria	o__Micrococcales	f__Microbacteriaceae	g__Parafrigoribacterium	0	0	0	0	0	0
d__Bacteria	p__Dependentiae	c__Babeliae	o__Babeliales	f__Babeliales	g__Babeliales	0	0	0	0	0	0
d__Bacteria	p__Firmicutes	c__Clostridia	o__Clostridiales	f__Clostridiaceae	g__Sarcina	0	0	0	0	0	0
d__Bacteria	p__Patescibacteria	c__Microgenomatia	o__Candidatus_Shapirobacteria	f__Candidatus_Shapirobacteria	g__Candidatus_Shapirobacteria	0	0	0	0	0	0
d__Bacteria	p__Proteobacteria	c__Gammaproteobacteria	o__Burkholderiales	f__Rhodocyclaceae	__	0	0.000104428	0	0	0	0
d__Bacteria	p__Firmicutes	c__Limnochordia	o__Limnochordales	f__Limnochordaceae	g__Limnochordaceae	0	0	0	0.000164096	0	0
d__Bacteria	p__Firmicutes	c__Clostridia	o__Peptostreptococcales-Tissierellales	f__Peptostreptococcaceae	g__Peptoclostridium	0	0	0	0	0	0
d__Archaea	p__Crenarchaeota	c__Bathyarchaeia	o__Bathyarchaeia	f__Bathyarchaeia	g__Bathyarchaeia	0	0	0	0	0	0
d__Archaea	p__Euryarchaeota	c__Thermococci	o__Methanofastidiosales	f__uncultured	g__uncultured	0	0	0	0	0	0
d__Bacteria	p__Actinobacteriota	c__Actinobacteria	o__Frankiales	f__Sporichthyaceae	g__Longivirga	0	0	0	0	0	0
d__Bacteria	p__Actinobacteriota	c__Actinobacteria	o__Micrococcales	f__Dermatophilaceae	g__Kineosphaera	0	0	0	0	0	0
d__Bacteria	p__Bacteroidota	c__Bacteroidia	o__Bacteroidales	f__Bacteroidales_UCG-001	g__Bacteroidales_UCG-001	0	0	0	0	0	0
d__Bacteria	p__Proteobacteria	c__Gammaproteobacteria	o__Burkholderiales	f__Comamonadaceae	g__Sphaerotilus	0	0	0	0	0	0
d__Bacteria	p__Spirochaetota	c__MVP-15	o__MVP-15	f__MVP-15	g__MVP-15	0	0	0	0	0	0
d__Bacteria	p__Desulfobacterota	c__Desulfuromonadia	o__Desulfuromonadia	f__Desulfuromonadaceae	__	0	0	0.00015004	0	0	0
d__Bacteria	p__Proteobacteria	c__Gammaproteobacteria	o__Xanthomonadales	f__Xanthomonadaceae	g__Arenimonas	0	0	0	0	0	0
d__Bacteria	p__Methylomirabilota	c__Methylomirabilia	o__Methylomirabilales	f__Methylomirabilaceae	g__Sh765B-TzT-35	0	0	0	0	0	0
d__Bacteria	p__Planctomycetota	c__Planctomycetes	o__Pirellulales	f__Pirellulaceae	g__Pir3_lineage	0	0	0	0	0	0
d__Bacteria	p__Proteobacteria	c__Alphaproteobacteria	o__Rhizobiales	f__Labraceae	g__Labrys	0	0	0	0	0	0
Unassigned	__	__	__	__	__	0	0	0	0.000140654	0	0
d__Bacteria	p__Actinobacteriota	c__Actinobacteria	o__Micrococcales	f__Intrasporangiaceae	g__Tetrasphaera	0	0	0	0	0	0
d__Bacteria	p__Firmicutes	c__Clostridia	o__Lachnospirales	f__Lachnospiraceae	__	0	0	0	0	0	0
d__Bacteria	p__Planctomycetota	c__Phycisphaerae	o__MSBL9	f__SG8-4	g__SG8-4	0	0	0	0	0	0
d__Bacteria	p__Proteobacteria	c__Alphaproteobacteria	o__Rhizobiales	f__Xanthobacteraceae	g__Afipia	0	0	0	0	0	0
d__Bacteria	p__Verrucomicrobiota	c__Chlamydiae	o__Chlamydiales	f__Criblamydiaceae	g__Criblamydiaceae	0	0	0	0	0	0
d__Bacteria	p__Bacteroidota	c__Bacteroidia	o__Bacteroidales	f__M2PB4-65_termite_group	g__M2PB4-65_termite_group	0	0	0	0	0	0
d__Bacteria	p__Firmicutes	c__Desulfitobacteriia	o__Desulfitobacteriales	f__Syntrophobotulaceae	g__Dehalobacter	0	0	0	0	0	0
d__Bacteria	p__Verrucomicrobiota	c__Chlamydiae	o__Chlamydiales	f__Parachlamydiaceae	__	0	0	0	0	0	0
d__Bacteria	p__Firmicutes	c__Clostridia	o__Clostridiales	f__Clostridiaceae	__	0.000122126	0	0	0	0	0
d__Bacteria	p__Patescibacteria	c__Parcubacteria	o__Candidatus_Vogelbacteria	f__Candidatus_Vogelbacteria	g__Candidatus_Vogelbacteria	0	0	0	0	0	0
d__Bacteria	p__Proteobacteria	c__Gammaproteobacteria	o__Burkholderiales	f__Rhodocyclaceae	g__Sulfuritalea	0	0	0	0	0	0
d__Bacteria	p__Actinobacteriota	c__Actinobacteria	o__Frankiales	f__Nakamurellaceae	g__Nakamurella	0	0	0	0	0	0
d__Bacteria	p__Actinobacteriota	c__Actinobacteria	o__Micrococcales	f__Microbacteriaceae	g__Leucobacter	0	0	0	0	0	0
d__Bacteria	p__Bacteroidota	c__Ignavibacteria	o__Ignavibacteriales	__	__	0	0	0	0	0	0
d__Bacteria	p__Proteobacteria	c__Alphaproteobacteria	o__Caulobacterales	f__Hyphomonadaceae	g__uncultured	0	0	0	0	0	0
d__Bacteria	p__Proteobacteria	c__Alphaproteobacteria	o__Reyranellales	f__Reyranellaceae	g__Reyranella	0	0	0	0	0	0
d__Bacteria	p__Proteobacteria	c__Alphaproteobacteria	o__Rickettsiales	f__Mitochondria	g__Mitochondria	0	0	0	0	0	0
d__Bacteria	p__Proteobacteria	c__Alphaproteobacteria	o__Sphingomonadales	f__Sphingomonadaceae	g__Sphingobium	0	0	0	0	0	0
d__Bacteria	p__Proteobacteria	c__Gammaproteobacteria	o__Gammaproteobacteria_Incertae_Sed	f__Unknown_Family	g__Acidibacter	0	0	0	0	0	0
d__Bacteria	p__Proteobacteria	c__Gammaproteobacteria	o__R7C24	f__R7C24	g__R7C24	0	0	0	0	0	0
d__Bacteria	p__Actinobacteriota	c__Actinobacteria	o__Propionibacteriales	f__Propionibacteriaceae	g__uncultured	0	0	0	0	0	0
d__Bacteria	p__Campilobacterota	c__Campylobacteria	o__Campylobacterales	f__Sulfurovaceae	g__Sulfurovum	0	0	0	0	0	0

d__Bacteria	p__Patescibacteria	c__Saccharimonadia	o__Saccharimonadales	f__LWQ8	g__LWQ8	0	0	0	0	0	0
d__Bacteria	p__Acidobacteriota	c__Acidobacteriae	o__GOUTB8	f__GOUTB8	g__GOUTB8	0	0	0	0	0	0
d__Bacteria	p__Acidobacteriota	c__Blastocatellia	o__DS-100	f__DS-100	g__DS-100	0	0	0	0	0	0
d__Bacteria	p__Firmicutes	c__Clostridia	o__Oscillospirales	f__Oscillospiraceae	g__Papillibacter	0	0	0	0	0	0
d__Bacteria	p__Patescibacteria	c__Parcubacteria	o__32-520	f__32-520	g__32-520	0	0	0	0	0	0
d__Bacteria	p__Proteobacteria	c__Alphaproteobacteria	o__Sphingomonadales	f__Sphingomonadaceae	g__Sphingomonas	0	0	0	0	0	0
d__Bacteria	p__Proteobacteria	c__Gammaproteobacteria	o__Burkholderiales	f__Comamonadaceae	g__Ottowia	0	0	0	0	0	0
d__Bacteria	p__Verrucomicrobiota	c__Lentisphaeria	o__Victivallales	f__vadinBE97	g__vadinBE97	0	0	0	0	0	0
d__Bacteria	p__Bacteroidota	c__Bacteroidia	o__Bacteroidales	f__Prolixibacteraceae	g__Mariniphaga	0	0	0	0	0	0
d__Bacteria	p__Firmicutes	c__Clostridia	o__Peptostreptococcales-Tissierellales	f__Peptostreptococcales-Tissierellales	g__W5053	0	0	0	0	0	0
d__Bacteria	p__Proteobacteria	c__Alphaproteobacteria	o__Caulobacterales	f__Hyphomonadaceae	__	0	0	0	0	0	0
d__Bacteria	p__Proteobacteria	c__Gammaproteobacteria	o__Gammaproteobacteria_Incertae_Sed	f__Unknown_Family	g__Candidatus_Berkiella	0	0	0	0	0	0
d__Bacteria	p__Acidobacteriota	c__Acidobacteriae	o__Solibacterales	f__Solibacteraceae	g__Candidatus_Solibacter	0	0	0	0	0.000100425	0
d__Bacteria	p__Desulfobacterota	c__Desulfovibrionia	o__Desulfovibrionales	f__Desulfovibrionaceae	g__uncultured	0	0	0	0	0	0
d__Bacteria	p__Actinobacteriota	c__Actinobacteria	o__Propionibacteriales	f__Propionibacteriaceae	g__Propionicimonas	0	0	0	0	0	0
d__Bacteria	p__Chloroflexi	c__KD4-96	o__KD4-96	f__KD4-96	g__KD4-96	0	0	0	0	0	0
d__Bacteria	p__Proteobacteria	c__Alphaproteobacteria	o__Rhizobiales	f__A0839	g__A0839	0	0	0	0	0	0
d__Bacteria	p__Sva0485	c__Sva0485	o__Sva0485	f__Sva0485	g__Sva0485	0	0	0	0	0	0
d__Bacteria	p__Acidobacteriota	c__Thermoanaerobaculia	o__Thermoanaerobaculales	f__Thermoanaerobaculaceae	g__Thermoanaerobaculum	0	0	0	0	0	0
d__Bacteria	p__Actinobacteriota	c__Acidimicrobiia	o__Microtrichales	f__Iamiaceae	g__Iamia	0	0	0	0	0	0
d__Bacteria	p__Actinobacteriota	c__Acidimicrobiia	o__Microtrichales	f__Microtrichaceae	g__IMCC26207	0	0	0	0	0	0
d__Bacteria	p__Patescibacteria	c__ABY1	o__Candidatus_Falkowbacteria	f__Candidatus_Falkowbacteria	g__Candidatus_Falkowbacteria	0	0	0	0	0	0
d__Bacteria	p__Patescibacteria	c__Parcubacteria	o__Parcubacteria	f__Parcubacteria	g__Parcubacteria	0	0	0	0	0	0
d__Bacteria	p__Proteobacteria	c__Gammaproteobacteria	o__PLTA13	f__PLTA13	g__PLTA13	0	0	0	0	0	0
d__Bacteria	p__Bacteroidota	c__Bacteroidia	o__Sphingobacteriales	f__AKYH767	g__AKYH767	0	0	0	0	0	0
d__Bacteria	p__Chloroflexi	c__SHA-26	o__SHA-26	f__SHA-26	g__SHA-26	0	0	0	0	0	0
d__Bacteria	p__Deinococcota	c__Deinococci	o__Deinococcales	f__Deinococcaceae	g__Deinococcus	0	0	0	0	0	0
d__Bacteria	p__Patescibacteria	c__ABY1	o__Candidatus_Magasanikbacteria	f__Candidatus_Magasanikbacteria	g__Candidatus_Magasanikbacteria	0	0	0	0	0	0
d__Bacteria	p__Patescibacteria	c__Parcubacteria	__	__	__	0	0	0	0	0	0
d__Bacteria	p__Planctomycetota	c__Planctomycetes	o__Pirellulales	f__Pirellulaceae	g__Blastopirellula	0	0	0	0	0	0
d__Bacteria	p__Planctomycetota	c__Planctomycetes	o__Planctomycetales	f__Schlesneriaceae	g__Planctopirus	0	0	0	0	0	0
d__Bacteria	p__Planctomycetota	c__Planctomycetes	o__Planctomycetales	f__Schlesneriaceae	g__Schlesneria	0	0	0	0	0	0
d__Bacteria	p__Proteobacteria	c__Alphaproteobacteria	o__Holosporales	f__Holosporaceae	g__uncultured	0	0	0	0	0	0
d__Bacteria	p__Proteobacteria	c__Gammaproteobacteria	o__Burkholderiales	f__Nitrosomonadaceae	g__Ellin6067	0	0	0	0	0	0
d__Bacteria	p__Proteobacteria	c__Gammaproteobacteria	o__Methylococcales	f__Methylococcaceae	g__Methyloparacoccus	0	0	0	0	0	0
d__Bacteria	p__Verrucomicrobiota	c__Verrucomicrobiae	o__Opitutales	f__Opitutaceae	g__Lacunisphaera	0	0	0	0	0	0
d__Bacteria	p__Cyanobacteria	c__Vampirivibrionia	o__Obscuribacterales	f__Obscuribacteraceae	g__Obscuribacteraceae	0	0	0	0	0	0
d__Bacteria	p__Verrucomicrobiota	c__Lentisphaeria	o__Oligosphaerales	f__Lenti-02	g__Lenti-02	0	0	0	0	0	0
d__Bacteria	p__Bdellovibrionota	c__Bdellovibrionia	o__Bdellovibrionales	f__Bdellovibrionaceae	g__Bdellovibrio	0	0	0	0	0	0
d__Bacteria	p__Firmicutes	c__BRH-c20a	o__BRH-c20a	f__BRH-c20a	g__BRH-c20a	0	0	0	0	0	0
d__Bacteria	p__Gemmatimonadot	c__MD2902-B12	o__MD2902-B12	f__MD2902-B12	g__MD2902-B12	0	0	0	0	0	0
d__Bacteria	p__Actinobacteriota	c__Thermoleophilia	o__Gaiellales	f__Gaiellaceae	g__Gaiella	0	0	0	0	0	0
d__Bacteria	p__Bacteroidota	c__Kryptonia	o__Kryptoniales	f__BSV26	g__BSV26	0	0	0	0	0	0
d__Bacteria	p__Dependentiae	c__Babeliae	o__Babeliales	f__Vermiphilaceae	g__Vermiphilaceae	0	0	0	0	0	0
d__Bacteria	p__NB1-j	c__NB1-j	o__NB1-j	f__NB1-j	g__NB1-j	0	0	0	0	0	0
d__Bacteria	p__Planctomycetota	c__Planctomycetes	o__uncultured	f__uncultured	g__uncultured	0	0	0	0	0	0
d__Bacteria	p__Proteobacteria	c__Alphaproteobacteria	o__Sphingomonadales	f__Sphingomonadaceae	g__Polymorphobacter	0	0	0	0	0	0
d__Bacteria	p__Proteobacteria	c__Gammaproteobacteria	o__Competibacterales	f__Competibacteraceae	g__Candidatus_Competibacter	0	0	0	0	0	0
d__Bacteria	p__Proteobacteria	c__Gammaproteobacteria	o__Diplorickettsiales	f__Diplorickettsiaceae	g__Rickettsiella	0	0	0	0	0	0
d__Bacteria	p__Acidobacteriota	c__Holophagae	o__Subgroup_7	f__Subgroup_7	g__Subgroup_7	0	0	0	0	0	0
d__Bacteria	p__Actinobacteriota	c__Actinobacteria	o__Propionibacteriales	f__Propionibacteriaceae	g__Propioniciclava	0	0	0	0	0	0
d__Bacteria	p__Bdellovibrionota	c__Bdellovibrionia	o__Bacteriovoracales	f__Bacteriovoracaceae	g__Peredibacter	0	0	0	0	0	0
d__Bacteria	p__Myxococcota	c__Polyangia	o__Polyangiales	f__Polyangiaceae	g__uncultured	0	0	0	0	0	0
d__Bacteria	p__Proteobacteria	c__Alphaproteobacteria	o__Rhizobiales	f__Beijerinckiaceae	__	0	0	0	0	0	0
d__Bacteria	p__Proteobacteria	c__Alphaproteobacteria	o__Rhodobacterales	f__Rhodobacteraceae	g__Roseobacter_clade_CHAB-I-5_lineage	0	0	0	0	0	0
d__Bacteria	p__Proteobacteria	c__Gammaproteobacteria	o__Burkholderiales	f__Aquaspirillaceae	g__Rivicola	0	0	0	0	0	0
d__Bacteria	p__Proteobacteria	c__Gammaproteobacteria	o__Xanthomonadales	f__Xanthomonadaceae	g__Pseudoxanthomonas	0	0	0	0	0	0
d__Bacteria	p__Chloroflexi	c__OLB14	o__OLB14	f__OLB14	g__OLB14	0	0	0	0	0	0
d__Bacteria	p__Patescibacteria	c__Microgenomatia	o__Candidatus_Levybacteria	f__Candidatus_Levybacteria	g__Candidatus_Levybacteria	0	0	0	0	0	0
d__Bacteria	p__Proteobacteria	c__Alphaproteobacteria	o__Azospirillales	f__Inquilinaceae	g__Inquilinus	0	0	0	0	0	0
d__Bacteria	p__Proteobacteria	c__Alphaproteobacteria	o__Caulobacterales	f__Caulobacteraceae	g__Caulobacter	0	0	0	0	0	0
d__Bacteria	p__Proteobacteria	c__Gammaproteobacteria	o__Burkholderiales	f__Nitrosomonadaceae	g__Nitrosomonas	0	0	0	0	0	0
d__Bacteria	p__Proteobacteria	c__Gammaproteobacteria	o__Coxiellales	f__Coxiellaceae	g__Coxiella	0	0	0	0	0	0
d__Bacteria	p__Proteobacteria	c__Gammaproteobacteria	o__uncultured	f__uncultured	g__uncultured	0	0	0	0	0	0
d__Bacteria	p__Acidobacteriota	c__Subgroup_18	o__Subgroup_18	f__Subgroup_18	g__Subgroup_18	0	0	0	0	0	0
d__Bacteria	p__Bacteroidota	c__Bacteroidia	o__Bacteroidales	f__p-251-o5	g__p-251-o5	0	0	0	0	0	0
d__Bacteria	p__Bacteroidota	c__Bacteroidia	o__Chitinophagales	f__uncultured	g__uncultured	0	0	0	0	0	0
d__Bacteria	p__Proteobacteria	c__Gammaproteobacteria	o__Diplorickettsiales	f__Diplorickettsiaceae	g__uncultured	0	0	0	0	0	0
d__Archaea	p__Halobacterota	c__Methanomicrobia	o__Methanomicrobiales	f__Methanocorpusculaceae	g__Methanocorpusculum	0	0	0	0	0	0
d__Bacteria	p__Firmicutes	c__Clostridia	o__Oscillospirales	f__Ethanoligenenaceae	g__Acetanaerobacterium	0	0	0	0	0	0
d__Bacteria	p__Proteobacteria	c__Alphaproteobacteria	o__Acetobacterales	f__Acetobacteraceae	g__Roseomonas	0	0	0	0	0	0
d__Bacteria	p__Verrucomicrobiota	c__Chlamydiae	o__Chlamydiales	f__cvE6	g__cvE6	0	0	0	0	0	0
d__Bacteria	p__Verrucomicrobiota	c__Verrucomicrobiae	o__Chthoniobacterales	f__Terrimicrobiaceae	g__Terrimicrobium	0	0	0	0	0	0
d__Bacteria	p__Actinobacteriota	c__Thermoleophilia	o__Solirubrobacterales	f__Solirubrobacteraceae	g__Conexibacter	0	0	0	0	0	0
d__Bacteria	p__Bacteroidota	c__Bacteroidia	o__Bacteroidales	f__Dysgonomonadaceae	__	0	0	0	0	0	0
d__Bacteria	p__Bacteroidota	c__Bacteroidia	o__Sphingobacteriales	f__S15A-MN91	g__S15A-MN91	0	0	0	0	0	0
d__Bacteria	p__Firmicutes	c__Clostridia	o__Clostridiales	f__Clostridiaceae	g__Clostridium_sensu_stricto_12	0	0	0	0	0	0
d__Bacteria	p__Proteobacteria	c__Gammaproteobacteria	o__Nitrosococcales	f__Nitrosococcaceae	g__Cl75cm.2.12	0	0	0	0	0	0
d__Bacteria	p__Bacteroidota	c__Bacteroidia	o__Flavobacteriales	f__NS9_marine_group	g__NS9_marine_group	0	0	0	0	0	0

d__Bacteria	p__Fibrobacterota	c__Fibrobacteria	o__Fibrobacterales	f__Fibrobacterales	g__BBMC-4	0	0	0	0	0	0
d__Bacteria	p__Firmicutes	c__Clostridia	o__Peptostreptococcales-Tissierellales	__	__	0	0	0	0	0	0
d__Bacteria	p__Myxococcota	c__Polyangia	o__Haliangiales	f__Haliangiaceae	g__Haliangium	0	0	0	0	0	0
d__Bacteria	p__Patescibacteria	c__Microgenomatia	o__Microgenomatia	f__Microgenomatia	g__Microgenomatia	0	0	0	0	0	0
d__Bacteria	p__Proteobacteria	c__Alphaproteobacteria	o__Rhizobiales	f__Rhizobiales_Incertae_Sedis	g__Bauldia	0	0	0	0	0	0
d__Bacteria	p__Proteobacteria	c__Gammaproteobacteria	__	__	__	0	0	0	0	0	0
d__Bacteria	p__Bacteroidota	c__Bacteroidia	o__Bacteroidales	f__Prolixibacteraceae	g__Roseimarinus	0	0	0	0	0	0
d__Bacteria	p__Bacteroidota	c__Bacteroidia	o__Bacteroidales	f__Rikenellaceae	g__SP3-e08	0	0	0	0	0	0
d__Bacteria	p__Bdellovibrionota	c__Oligoflexia	o__Oligoflexales	f__uncultured	g__uncultured	0	0	0	0	0	0
d__Bacteria	p__Calditrichota	c__Calditrichia	o__Calditrichales	f__Calditrichaceae	g__SM23-31	0	0	0	0	0	0
d__Bacteria	p__Chloroflexi	c__Dehalococcoidia	o__Dehalococcoidales	f__Dehalococcoidaceae	g__Dehalogenimonas	0	0	0	0	0	0
d__Bacteria	p__Firmicutes	c__Clostridia	o__Lachnospirales	f__Lachnospiraceae	g__Epulopiscium	0	0	0	0	0	0
d__Bacteria	p__MBNT15	c__MBNT15	o__MBNT15	f__MBNT15	g__MBNT15	0	0	0	0	0	0
d__Bacteria	p__Myxococcota	c__bacteriap25	o__bacteriap25	f__bacteriap25	g__bacteriap25	0	0	0	0	0	0
d__Bacteria	p__Planctomycetota	c__Planctomycetes	o__Pirellulales	f__Pirellulaceae	g__Pir2_lineage	0	0	0	0	0	0
d__Bacteria	p__Proteobacteria	c__Alphaproteobacteria	o__Sphingomonadales	f__Sphingomonadaceae	__	0	0	0	0	0	0
d__Bacteria	p__Proteobacteria	c__Gammaproteobacteria	o__Burkholderiales	__	__	0	0	0	0	0	0
d__Bacteria	p__Proteobacteria	c__Gammaproteobacteria	o__Xanthomonadales	f__Rhodanobacteraceae	g__Chiayiivirga	0	0	0	0	0	0
d__Bacteria	p__Chloroflexi	c__Anaerolineae	__	__	__	0	0	0	0	0	0
d__Bacteria	p__Firmicutes	c__Bacilli	o__Erysipelotrichales	f__Erysipelotrichaceae	g__Erysipelothrix	0	0	0	0	0	0
d__Bacteria	p__Firmicutes	c__Negativicutes	o__Veillonellales-Selenomonadales	f__Veillonellaceae	g__Veillonella	0	0	0	0	0	0
d__Bacteria	p__Proteobacteria	c__Gammaproteobacteria	o__Burkholderiales	f__Hydrogenophilaceae	g__Thiobacillus	0	0	0	0	0	0
d__Bacteria	p__Proteobacteria	c__Gammaproteobacteria	o__Gammaproteobacteria_Incertae_Sed	f__Unknown_Family	g__Unknown_Family	0	0	0	0	0	0
d__Bacteria	p__Verrucomicrobiota	c__Verrucomicrobiae	o__Pedosphaerales	f__Pedosphaeraceae	__	0	0	0	0	0	0
